# A randomized, open-label, adaptive, proof-of-concept clinical trial of modulation of host thromboinflammatory response in patients with COVID-19: the DAWn-Antico study

**DOI:** 10.1186/s13063-020-04878-y

**Published:** 2020-12-09

**Authors:** T. Vanassche, M. M. Engelen, Q. Van Thillo, J. Wauters, J. Gunst, C. Wouters, C. Vandenbriele, S. Rex, L. Liesenborghs, A. Wilmer, P. Meersseman, G. Van den Berghe, D. Dauwe, G. Verbeke, M. Thomeer, T. Fivez, D. Mesotten, D. Ruttens, L. Heytens, I. Dapper, S. Tuyls, B. De Tavernier, P. Verhamme, DAWn consortium members, Iwein Gyselinck, Laure-Anne Teuwen, Vincent Geldhof, Ewout Landeloos, Tatjana Geukens, Helga Ceunen, Barbara Debaveye, Caroline Devooght, Anna Ockerman, Veerle Servaes, Ann Belmans

**Affiliations:** 1 Center for Molecular and Vascular Biology, KU Leuven Department of Cardiovascular Sciences, KU Leuven, Leuven, Belgium; 2 Department of Cardiovascular Sciences, University Hospitals Leuven, Leuven, Belgium; 3 Center for Cancer Biology, VIB, Leuven, Belgium; 4 Department of General Internal Medicine, Medical Intensive Care Unit, University Hospitals Leuven, Leuven, Belgium; 5 Clinical Department and Laboratory of Intensive Care Medicine, Department of Cellular and Molecular Medicine, KU Leuven, Leuven, Belgium; 6 Pediatric Rheumatology, University Hospitals Leuven, Leuven, Belgium; 7 Laboratory of Adaptive Immunology & Immunobiology, Department of Microbiology and Immunology, KU Leuven, Leuven, Belgium; 8 Department of Anesthesiology, University Hospitals Leuven, Leuven, Belgium; 9 REGA Institute, KU Leuven, Leuven, Belgium; 10 Interuniversity Institute for Biostatistics and statistical Bioinformatics (I-BioStat), KU Leuven, Leuven, and Hasselt University (UHasselt), Hasselt, Belgium; 11 Department of Respiratory Medicine, Ziekenhuis Oost-Limburg, Genk, Belgium; 12 Department of Medicine and Life Sciences, Hasselt University, Diepenbeek, Belgium; 13 Department of Anaesthesiology, Intensive Care, Emergency Medicine and Pain Therapy, Ziekenhuis Oost-Limburg, Genk, Belgium; 14 Department of Anesthestiology, GZA hospital group, Antwerp, Belgium; 15 Emergency Medicine and Intensive Care, GZA hospital group, Antwerp, Belgium; 16 Respiratory Medicine, GZA hospital group, Antwerp, Belgium

**Keywords:** COVID-19, SARS-CoV-2, Low molecular weight heparins, Aprotinin, Anakinra, Thromboinflammatory response, Thrombosis, Inflammation

## Abstract

**Background:**

The peak of the global COVID-19 pandemic has not yet been reached, and many countries face the prospect of a second wave of infections before effective vaccinations will be available. After an initial phase of viral replication, some patients develop a second illness phase in which the host thrombotic and inflammatory responses seem to drive complications. Severe COVID-19 disease is linked to high mortality, hyperinflammation, and a remarkably high incidence of thrombotic events. We hypothesize a crucial pathophysiological role for the contact pathway of coagulation and the kallikrein-bradykinin pathway. Therefore, drugs that modulate this excessive thromboinflammatory response should be investigated in severe COVID-19.

**Methods:**

In this adaptive, open-label multicenter randomized clinical trial, we compare low molecular weight heparins at 50 IU anti-Xa/kg twice daily—or 75 IU anti-Xa twice daily for intensive care (ICU) patients—in combination with aprotinin to standard thromboprophylaxis in hospitalized COVID-19 patients. In the case of hyperinflammation, the interleukin-1 receptor antagonist anakinra will be added on top of the drugs in the interventional arm. In a pilot phase, the effect of the intervention on thrombotic markers (D-dimer) will be assessed. In the full trial, the primary outcome is defined as the effect of the interventional drugs on clinical status as defined by the WHO ordinal scale for clinical improvement.

**Discussion:**

In this trial, we target the thromboinflammatory response at multiple levels. We intensify the dose of low molecular weight heparins to reduce thrombotic complications. Aprotinin is a potent kallikrein pathway inhibitor that reduces fibrinolysis, activation of the contact pathway of coagulation, and local inflammatory response. Additionally, aprotinin has shown in vitro inhibitory effects on SARS-CoV-2 cellular entry. Because the excessive thromboinflammatory response is one of the most adverse prognostic factors in COVID-19, we will add anakinra, a recombinant interleukin-1 receptor antagonist, to the regimen in case of severely increased inflammatory parameters. This way, we hope to modulate the systemic response to SARS-CoV-2 and avoid disease progressions with a potentially fatal outcome.

**Trial registration:**

The EU Clinical Trials Register 2020-001739-28. Registered on April 10, 2020.

**Supplementary information:**

The online version contains supplementary material available at 10.1186/s13063-020-04878-y.

## Administrative information

**Table:** 

Title {1}	A randomized, open-label, adaptive, proof-of-concept clinical trial of modulation of host thromboinflammatory response in patients with COVID-19: the DAWn-Antico study
Trial registration {2a and 2b}.	EU Clinical Trials Register; 2020-001739-28. Registered on 2020-04-10.
Protocol version {3}	2020-05-05, DAWn-AntiCo v2.1 This study is part of Direct Antivirals Working against nCoV (DAWn) studies.
Funding {4}	This study is funded by Life Sciences Research Partners (LSRP), Research Foundation - Flanders (FWO) project G0G4720N and the COVID-19 fund of the KU Leuven.
Author details {5a}	^1^ Center for Molecular and Vascular Biology, KU Leuven Department of Cardiovascular Sciences, KU Leuven, Belgium ^2^ Center for Cancer Biology, VIB, KU Leuven, Leuven, Belgium ^3^ Department of General Internal Medicine, Medical Intensive Care Unit, University Hospitals Leuven, Leuven, Belgium ^4^ Clinical Department and Laboratory of Intensive Care Medicine, Department of Cellular and Molecular Medicine, KU Leuven, Leuven, Belgium ^5^ Pediatric Rheumatology, University Hospitals Leuven, Leuven, Belgium ^6^ Department of Anesthesiology, University Hospitals Leuven, Leuven, Belgium and Department of Cardiovascular Sciences, KU Leuven, Leuven, Belgium ^7^ Department of Microbiology, Immunology and Transplantation, Rega Institute, Virology and Chemotherapy, Molecular Vaccinology & Vaccine Discovery, KU Leuven, Leuven, Belgium ^8^ Department of Public Health and Primary Care, KU Leuven, Leuven, Belgium ^9^ Interuniversity Institute for Biostatistics and statistical Bioinformatics (I-BioStat), KU Leuven, Leuven, and Hasselt University (UHasselt), Hasselt, Belgium. ^10^ Department of Respiratory Medicine, Ziekenhuis Oost-Limburg, Genk, Belgium ^11^ Department of Medicine and Life Sciences, Hasselt University, Diepenbeek, Belgium ^12^ Department of Intensive Care, Ziekenhuis Oost-Limburg, Genk, Belgium ^13^ Department of Intensive Care, GZA hospital group, Antwerp, Belgium ^14^ Department of Respiratory Medicine, GZA hospital group, Antwerp, Belgium ^15^ Department of Emergency Care, GZA hospital group, Antwerp, Belgium
Name and contact information for the trial sponsor {5b}	Caroline Devooght, UZ Leuven.Herestraat 49, 3000 Leuven, Belgium.Contact: caroline.devooght@uzleuven.be
Role of sponsor {5c}	The sponsor was represented in the steering committee. They had full insight into the design, collection, management, analysis and interpretation of data.

## Introduction

### Background and rationale {6a}

COVID-19 (coronavirus disease-19) has caused a pandemic with an enormous burden for healthcare systems worldwide [1]. In the absence of immunity, a large part of the world’s population remains at risk for infection during a second wave until large-scale vaccination programs become available. Although SARS-CoV-2 causes little symptoms in many patients, a subset of patients develops an aggressive disease course characterized by severe pulmonary involvement and a high mortality [2]. In these patients with severe COVID-19 disease, hyperinflammation is prominent with an important involvement of the kallikrein-bradykinin and contact pathway [3–6]. Internalization of SARS-CoV-2 (severe acute respiratory syndrome coronavirus 2) involves binding to the ACE2 (angiotensin-converting enzyme 2) receptor, which is abundantly expressed in the respiratory epithelium [7, 8]. ACE2 has local anti-inflammatory functions by inactivating kallikreins and bradykinins. A loss of ACE2 function can lead to an overactivation of the kallikrein-bradykinin pathway with pulmonary inflammation and edema [9].

**Fig. 1:**
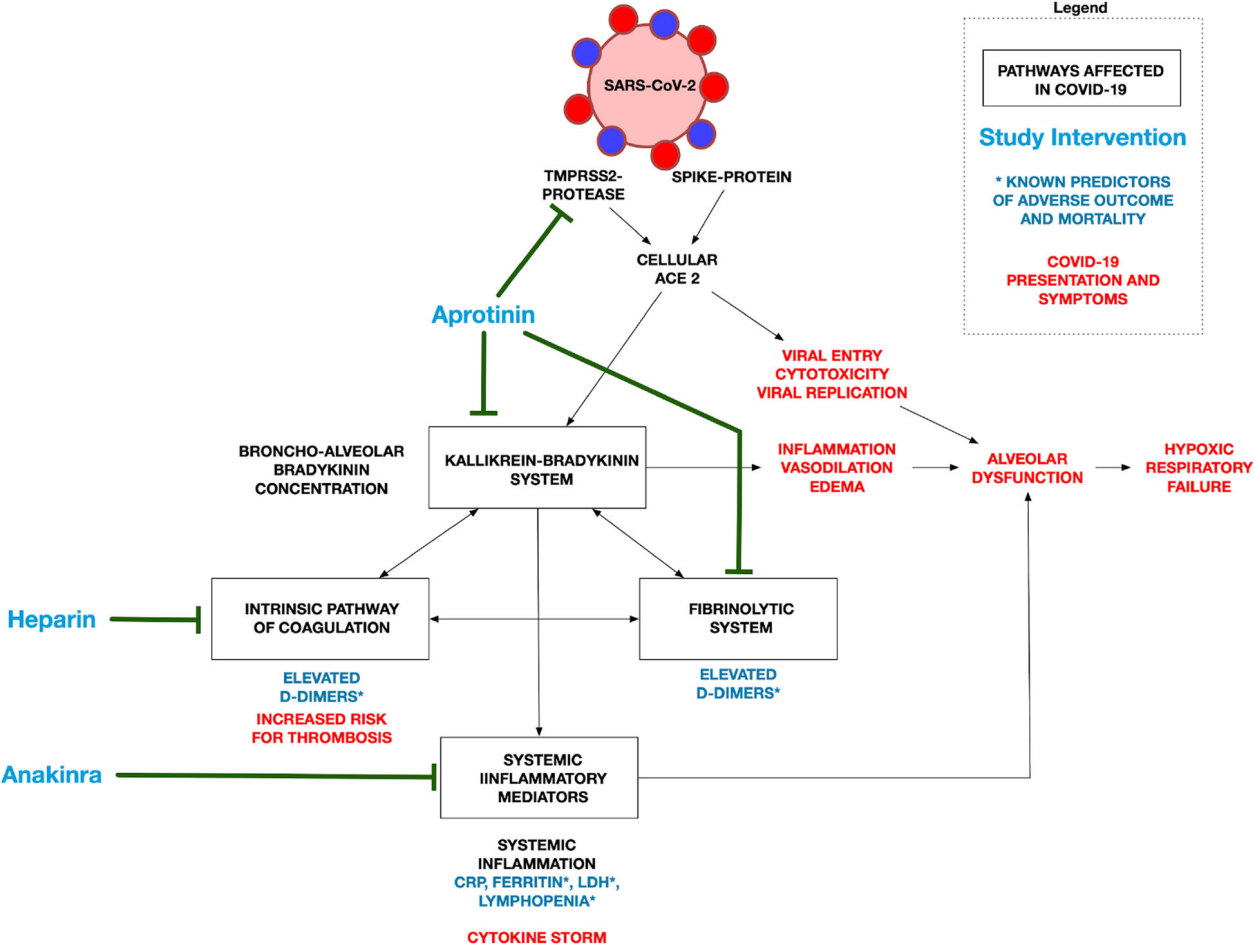
Effect of the investigated drugs on the pathways that are implicated in COVID-19

Moreover, the kallikrein-bradykinin system is closely linked to the contact pathway of the coagulation system via activation of factor XII, leading to local fibrin deposition, as well as diffuse intravascular coagulation (Fig. 1) [10]. D-dimers, which are a marker for hyperfibrinolysis and high ferritin and LDH levels, predict a poor outcome in COVID-19 [2, 3]. There is also increasing evidence that drugs influencing these pathways may lead to improved outcomes [11–13]. These observations provide a proof-of-concept for our current trial in which we want to target this presumably excessive thromboinflammatory response with aprotinin, low molecular weight heparins (LMWHs), and anakinra.

Specific inhibitors of the bradykinin-receptor are currently not available. However, kallikrein activity is strongly suppressed by aprotinin with a much higher affinity than tranexamic acid [14]. Aprotinin can also inhibit the protease activity of TMPRSS2 [15, 16]. Treatment of hypercoagulability, a hallmark of severe COVID-19, with LMWH is associated with increased survival compared to patients who did not receive anticoagulation in a recent study [12, 13]. Anakinra can modify hyperinflammation by inhibition of IL-1 and has been associated with potential benefit in non-randomized studies in patients with severe COVID-19 [17, 18].

Taken together, our trial consists of a unique approach based on the pathophysiology of COVID-19. There is thus a rationale for testing drugs explicitly targeting the thromboinflammatory response on multiple levels and evaluating the effect on biochemical markers, such as D-dimers, and clinical outcome.

### Objectives {7}

The study objectives are adapted from the WHO master protocol that was proposed to streamline interventional studies in patients with COVID-19 [19, 20]. The overall objective of the DAWn study is to evaluate the clinical efficacy and safety of investigational therapeutic agents relative to the standard of care in patients hospitalized with COVID-19. Additionally, the effect of interventions targeting the host response modulation will be evaluated separately in a pilot phase by a primary laboratory endpoint (change in thromboinflammatory biomarkers), and clinical outcomes will be evaluated as a secondary outcome.

Secondary objectives are to evaluate the clinical efficacy of different investigational therapeutics as compared to one another or the control arm.

All DAWn studies will investigate the same primary and secondary endpoints, listed in the full protocol available in an appendix to this paper. The primary endpoint is time to sustained clinical improvement, defined as a sustained 2-point improvement on the 7-rank WHO clinical status scale (see appendix). The essential endpoints are summarized below:

- Clinical severity defined as the time from day 0 to sustained clinical improvement (improvement of > 2 points vs. the highest value of days 0 and 1 and sustained for at least 3 days) or live discharge, whichever comes first
- Host thromboinflammatory status (DAWn-Antico): D-dimer levels, CRP, ferritin, LDH levels, and interleukin profile
- Oxygenation: incidence, duration, and oxygenation-free days
- Mechanical ventilation: incidence, duration, and ventilator-free days
- Hospitalization: duration, time to live after discharge
- Mortality (day 15, day 28, time to death)
- Safety of the interventions: serious adverse events (SAEs) and adverse events (AEs) graded as severe, discontinuation or temporary suspension of drug administration (for any reason), changes in laboratory parameters (complete blood count, renal and liver function)

### Trial design {8}

This study is part of the larger DAWn consortium. The DAWn-Antico study is an adaptive, randomized, parallel-group open-label multicenter clinical trial to evaluate the safety and efficacy of promising agents in hospitalized adult patients diagnosed with COVID-19. The study is a phase 2 proof-of-concept multicenter trial program in patients with COVID-19. The adaptive study design of DAWn allows for the addition of new treatment and strata during the study based on the most updated information.

**Fig. 2:**
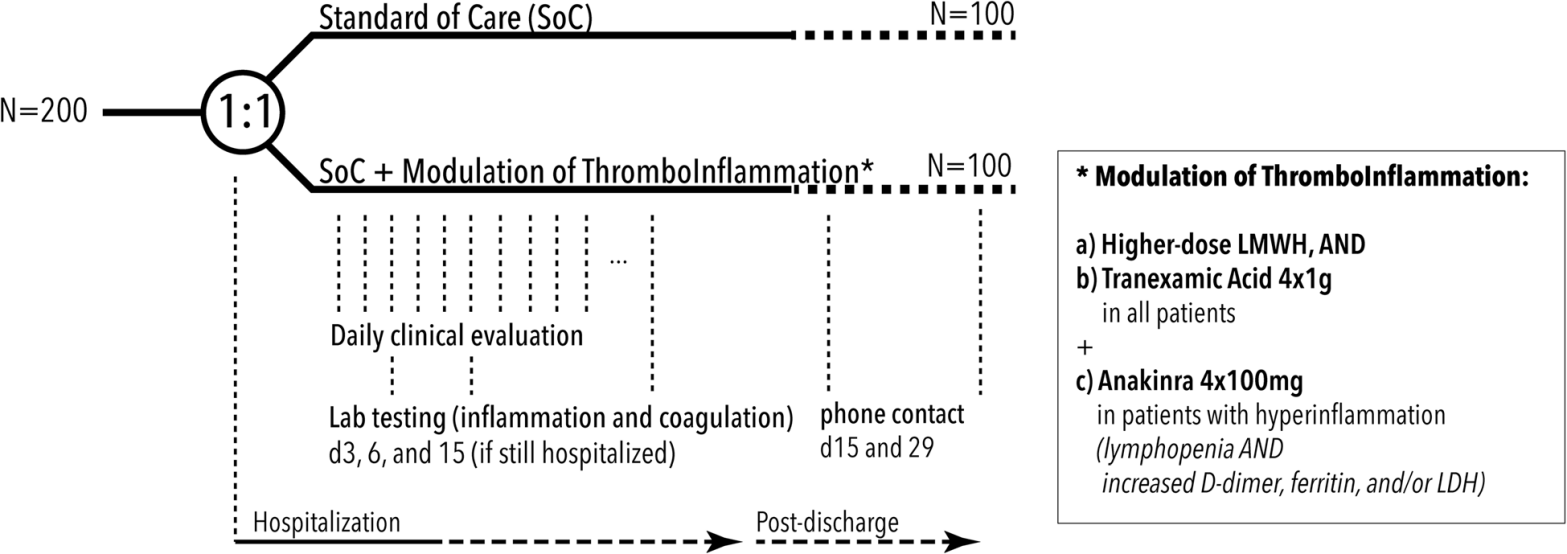
Trial design

With a 2:1 randomization, the DAWn-Antico study evaluates standard thromboprophylaxis (low molecular weight heparin (LMWH) at 50 IU/kg once daily in hospitalized patients and twice daily in patients on intensive care units) with a multi-step antithrombotic and anti-inflammatory strategy (Fig. 2). This strategy consists of the following:

A. A higher prophylactic dose of subcutaneous LMWH: 50 IU/kg twice daily in hospitalized patients and 75 IU/kg twice daily in patients on intensive care units from day 0 to 15, AND
B. Aprotinin 2 × 10^6^ KIE 4 times per day as intravenous (IV) infusion from day 0 to 3 for a total of 72 h (3 × 4 doses)
C. Additionally, patients with biochemical signs of hyperinflammation at baseline or during follow-up will receive add-on interleukin-1 receptor blockage by anakinra 100 mg 4× daily. Hyperinflammation is defined as an absolute lymphocyte count < 1000 cells/mL and two of the following: (1) ferritin > 800 ng/mL, (2) LDH > 400 U/L, or (3) D-dimers > 1000 ng/mL.

DAWN-AntiCo will start with a pilot phase powered to detect the biochemical response and will continue to detect the effect on clinical outcomes. In this trial, a superiority hypothesis will be tested with a two-sided type I error rate of 0.05. Secondary hypotheses will be tested in a non-hierarchical way. The statistical analysis plan will be finalized before the database lock.

## Methods: participants, interventions, and outcomes

### Study setting {9}

Study participants will be recruited from different hospitals in Belgium: the University Hospitals Leuven, Ziekenhuis Oost-Limburg Genk, and GZA Hospital Group Antwerp.

### Eligibility criteria {10}

In short, participants eligible for inclusion are hospitalized, adult patients with confirmed and severe COVID-19. Exclusion criteria are both general and drug-specific. Inclusion and exclusion criteria are summarized below; the fully detailed criteria are listed in the appendix.

#### Inclusion criteria

- Male or non-pregnant female adult ≥ 18 years of age at the time of enrolment
- Confirmed diagnosis of SARS-CoV-2 infection, defined as *either*:
  - SARS-CoV-2 PCR positive within 72 h before randomization
    *or*
  - Respiratory infection symptoms (fever, cough, dyspnea, desaturation) and typical findings on chest CT scan and absence of other plausible diagnoses.
    In patients without PCR-confirmed diagnosis at inclusion, all efforts will be made to confirm definite SARS-CoV-2 infection (e.g., PCR on bronchial aspirate, PCR on BAL fluid or serologic testing). Participants who—despite all efforts—do not have a confirmed diagnosis of COVID-19 will be excluded from the analysis.
- Illness of any duration and at least one of the following:
  - Radiographic infiltrates by imaging (chest x-ray, CT scan, etc.) *or*
  b. Clinical assessment (evidence of rales/crackles on lung auscultation) AND
    SpO2 ≤ 94% on room air *or*
  c. Requiring mechanical ventilation and/or supplemental oxygen.

#### Exclusion criteria

1. ALT/AST > 8 times the upper limit of normal
2. Pregnancy or breastfeeding
3. Known hypersensitivity to any study medication
4. Any medical condition which would impose an unacceptable safety hazard by participation in the study
5. Study drug-specific exclusion criteria:
  For aprotinin
  Known active thromboembolic disease (unprovoked venous thromboembolism (VTE), VTE in the last 3 months, myocardial infarction or coronary stenting in the last 6 months, ischemic stroke in the last 6 months)
  Renal insufficiency with creatinine clearance (CrCl) < 30 mL/min or renal replacement therapy
  For high-prophylactic dose of LMWH
  Active bleeding, a history of intracranial bleeding, or a recent (< 3 m) gastrointestinal (GI) bleeding requiring transfusion and/or intervention, recent surgery in the central nervous system
  Renal insufficiency with CrCl < 20 mL/min or renal replacement therapy
  Blood platelet count < 30,000/μL
  Increased risk of bleeding as judged by the investigator
  Need for therapeutic anticoagulation (known active thromboembolic diseases, atrial fibrillation, mechanical prosthetic heart valve…)
  Active bacterial endocarditis
  Chronic alcoholism
  For anakinra
  Impairment of cardiac function defined as severe heart failure, unstable angina pectoris, myocardial infarction within 6 months before enrollment, ventricular arrhythmia requiring treatment or intervention
  Severe renal dysfunction (CrCl ≤ 20 mL/min) or renal replacement therapy
  Uncontrolled hypertension (persistent SBP > 180 mmHg, or DBP > 110 mmHg)
  Clinical suspicion of latent tuberculosis or severe bacterial surinfection
  Known hypersensitivity to proteins produced by *E. coli*

### Who will take informed consent? {26a}

Potential participants are screened on the emergency ward or upon arrival at the COVID-ward. Accordingly, an emergency physician or a supervising physician at the COVID-ward who is trained in the protocol will first assess the patient’s interest in the study, if necessary through the legal representative. If there is an interest in participating, the investigators of the DAWn-study team again check the eligibility criteria and contact the patient or legal representative to provide more information and obtain informed consent. When signed informed consent is not permitted because of safety regulations related to the prevention of the transmission of SARS-CoV-2, the ethical committee approved the use of verbal informed consent. The consent form is read and illustrated by the investigator on the phone, and the patient or legal representative gives verbal consent which will be documented in the medical records, all in the presence of a witness. Signed informed consent shall then be obtained as soon as permitted based on safety regulations to prevent the transmission of SARS-CoV-2. The process for obtaining and documenting initial and continued informed consent from potential trial participants will be conducted following the International Conference on Harmonization of Technical Requirements for Registration of Pharmaceuticals for Human Use–Good Clinical Practice or ICH-GCP E6(R2), applicable regulatory requirements, and internal standard operating procedures (SOPs).

### Additional consent provisions for collection and use of participant data and biological specimens {26b}

Data can be reviewed by an audit group or Ethics Committee to verify the quality of the trial. Encoded trial data may be sent to other EU and non-EU countries for review by health authorities, Ethics Committee, external researchers, and the sponsor of the trial. The results of the trial will be used to answer the scientific questions of the trial. In addition, the sponsor can use the participant’s data obtained from this trial, in connection with other research and development activities. Any additional research outside of the trial must be approved by a Belgian recognized Ethics Committee.

Since scientific progress in this area is constant, the remainders of the patient’s biological samples will be kept for 30 years. The samples will be used for future research, to better understand the disease, its treatment and the responses to this treatment, and the admitted study drugs. Any future research, additional to what is described above, may only be conducted according to the legislation on the use of human tissue material and with the approval of a Belgian recognized Ethics Committee. As a general rule, the participant will be asked to sign an additional informed consent form in which the additional research is specified.

## Interventions

### Explanation for the choice of comparators {6b}

Following the guidelines of the Belgian Society on Thrombosis and Haemostasis and Sciensano, we use a higher than usual dose of thromboprophylaxis in the control arm [21]. ICU patients receive a dose of 50 IU anti-Xa/kg LMWH twice daily; ICU patients with a creatinine clearance below 30 mL/min and patients admitted to the wards receive a dose of 50 IU anti-Xa/kg LMWH once daily.

### Intervention description {11a}

Interventions (Fig. 2) consist of aprotinin on top of a higher dose of thromboprophylaxis. Anakinra will be added as soon as signs of hyperinflammation are observed.

#### Higher dose thromboprophylaxis

Patients randomized to the intervention arm will receive LMWH at 50 IU/kg (e.g., enoxaparin 0.5 mg/kg) twice daily, with a minimum of 4000 IU (e.g., 40 mg enoxaparin) twice daily, in non-ICU patients and 75 IU/kg (e.g., enoxaparin 0.75 mg/kg) twice daily—with a minimum of 4000 IU (e.g., enoxaparin 40 mg) twice daily—in ICU patients. The total daily dose will be reduced by 50% in patients with a CrCl < 30 mL/min (once daily instead of twice-daily administration). In patients who develop severe renal dysfunction (CrCl < 15 mL/min) or who require renal replacement therapy, the administration of LMWH should be interrupted until CrCl returns ≥30 mL/min. In patients who develop a CrCl < 15 mL/min, choice and dose of thromboprophylactic therapy is left to the discretion of the investigator. Options may include unfractionated heparin in a continuous IV infusion with a target activated partial thromboplastin time (aPTT) 40–60 s or a target anti-Xa of 0.2–0.4 U/mL, and with routine follow-up of aPTT or anti-Xa as per local practice. In patients receiving aprotinin (first 72 h), aPTT and ACT should not be used to monitor heparin effects, and the use of anti-Xa levels is suggested for heparin monitoring.

#### Aprotinin

Additionally, to suppress hyperfibrinolysis and the bradykinin-kallikrein pathway, patients will receive aprotinin. Aprotinin will be administered as an IV infusion of 2 MIE over 30 min four times per day from day 0 to day 3. Before the first administration, a test dose of 1 mL of a 50-mL vial will be given, with control of vital parameters for 20 min, before proceeding to full study dose. Administration of aprotinin will be at 6-h intervals, with the first administration together with the first administration of study LMWH. Further details on administration are disclosed in the full protocol affixed.

#### Treatment of hyperinflammation

Patients in the intervention arm will be monitored for hyperinflammation. If, at any point during the study, the patient develops signs of hyperinflammation, treatment with anakinra will be started if there are no contraindications (see the “Exclusion criteria” section).

Presence of hyperinflammation is defined as follows:

- Absolute lymphocyte count < 1000 cells/mL
- Two of the following: (i) ferritin > 800 ng/mL, (ii) LDH > 400 U/L, or (iii) D-dimers > 1000 ng/mL

These lab values have been identified as independent predictors of adverse outcome in patients with severe COVID-19 [2, 4, 22, 23].

Anakinra will be administered by IV infusion at a total dose of 400 mg per day, divided into 4 doses 100 mg IV every 6 h. Anakinra treatment will continue for 15 days, i.e., days 1 to 15. Before administration, the full content of the prefilled, single-use syringe (anakinra 100 mg) will be diluted in 100 mL saline. The IV administration of anakinra has to occur immediately after the preparation over an infusion period of 60 min. Full instructions for the preparation of anakinra are available in the IMP manual. Conditions for storage and injection are described in the attached protocol.

### Criteria for discontinuing or modifying allocated interventions {11b}

Circumstances requiring premature treatment interruption or discontinuation of the trial include but are not limited to (1) safety concerns related to IMP or unacceptable intolerability, (2) trial participation while in violation of the inclusion and/or exclusion criteria, (3) pregnancy or intention of becoming pregnant, (4) thrombotic complication, (5) bleeding complication, and (6) seizures. In patients receiving anakinra who develop ventricular arrhythmia requiring intervention, uncontrolled hypertension, or severe renal dysfunction or renal replacement therapy, anakinra infusion will be stopped.

In any such case of early trial termination and/or treatment interruption/discontinuation, the investigator will continue to monitor the participant’s condition closely and ensure adequate medical care and follow-up.

### Strategies to improve adherence to interventions {11c}

For each drug, SOPs were created. Detailed information on administration is provided within the hospital’s electronic system and is displayed when nurses administer the drugs. Permanent remote assistance is available as investigators are on standby 24 h a day, 7 days a week.

Investigators and clinical trial assistants (CTAs) will monitor drug administration daily. Remarks or problems with administration, which did not need telephonic assistance, can be marked via the electronic system. Per protocol, laboratory tests will be evaluated daily. Based on these assessments, some patients will be started on anakinra when criteria are met.

### Relevant concomitant care permitted or prohibited during the trial {11d}

There are currently no approved treatments for COVID-19. Patients will receive the standard of care as continuously updated by national and international guidance. Because the intervention of DAWn-AntiCo is independent of the background antiviral therapy, any antiviral therapy is allowed, including other investigational agents. Thus, concomitant participation in a study with antiviral agents does not exclude a patient from randomization within DAWn-AntiCo.

### Provisions for post-trial care {30}

As per European legislation, the sponsor has a full insurance that covers the costs of potential harms.

### Outcomes {12}

The study outcomes are based on the WHO master protocol, which has been proposed for all COVID-related research by the WHO [19, 20]. The ordinal scale below has also been used in multiple other COVID-19 studies [24, 25]. All outcomes will be presented overall as well as separately for patients with mild/moderate vs. severe disease at baseline.

Pilot phase of DAWn-Antico:

We will conduct a pilot phase with a pilot phase-specific primary endpoint analysis of D-dimer levels measured on day 6.

#### Primary outcome

The primary outcome is the time from day 0 to sustained clinical improvement or live discharge, whichever comes first, whereby a sustained clinical improvement is defined as an improvement of > 2 points vs. the highest value of days 0 and 1 and sustained for at least 3 days.

Clinical status of the subject at day 15 (on a 7-point ordinal scale):

1. Not hospitalized, no limitations on activities
2. Not hospitalized, limitation on activities
3. Hospitalized, not requiring supplemental oxygen
4. Hospitalized, requiring supplemental oxygen
5. Hospitalized, on non-invasive ventilation or high flow oxygen devices
6. Hospitalized, on invasive mechanical ventilation or extracorporeal membrane oxygenation (ECMO)
7. Death

#### Secondary outcomes

Secondary outcomes include clinical outcomes and markers of thromboinflammation at predefined time points. A summary is listed below; the detailed list is provided in the full protocol attached.

- Clinical status and time to clinical improvement
- Mortality (day 15, day 28, time to death)
- Duration of supplemental oxygen, mechanical ventilation, hospitalization, ICU stay, time to live weaning, and time to live discharge
- Usages and doses of rescue anti-inflammatory therapy (such as corticosteroids, IL-1 or IL-6 blockers—non-limitative)
- Adverse events
- Standard laboratory values
- Markers of hyperinflammation (CRP, ferritin, LDH, interleukin profile)
- Markers of thrombotic activation and kallikrein-bradykinin activation (D-dimers, fibrinogen, PT, aPTT, C1-inhibitor, factor XII)
- Markers of adrenal function (cortisol, ACTH, albumin/transcortin)
- Combined cardiac endpoint during hospitalization
- Incidence of thrombotic events and major bleeding complications during hospitalization as per ISTH criteria

### Overall long-term exploratory outcome

- Five to 7 weeks post-discharge, patients are invited to the respiratory clinic for (lung) functional and radiological evaluation if possible.
- A telephone call will be done on D90 post-admission for survival status evaluation.

### Participant timeline {13}

A schedule of enrolment, interventions, assessments, and visits for participants is shown in Table 1.

**Table 1:**
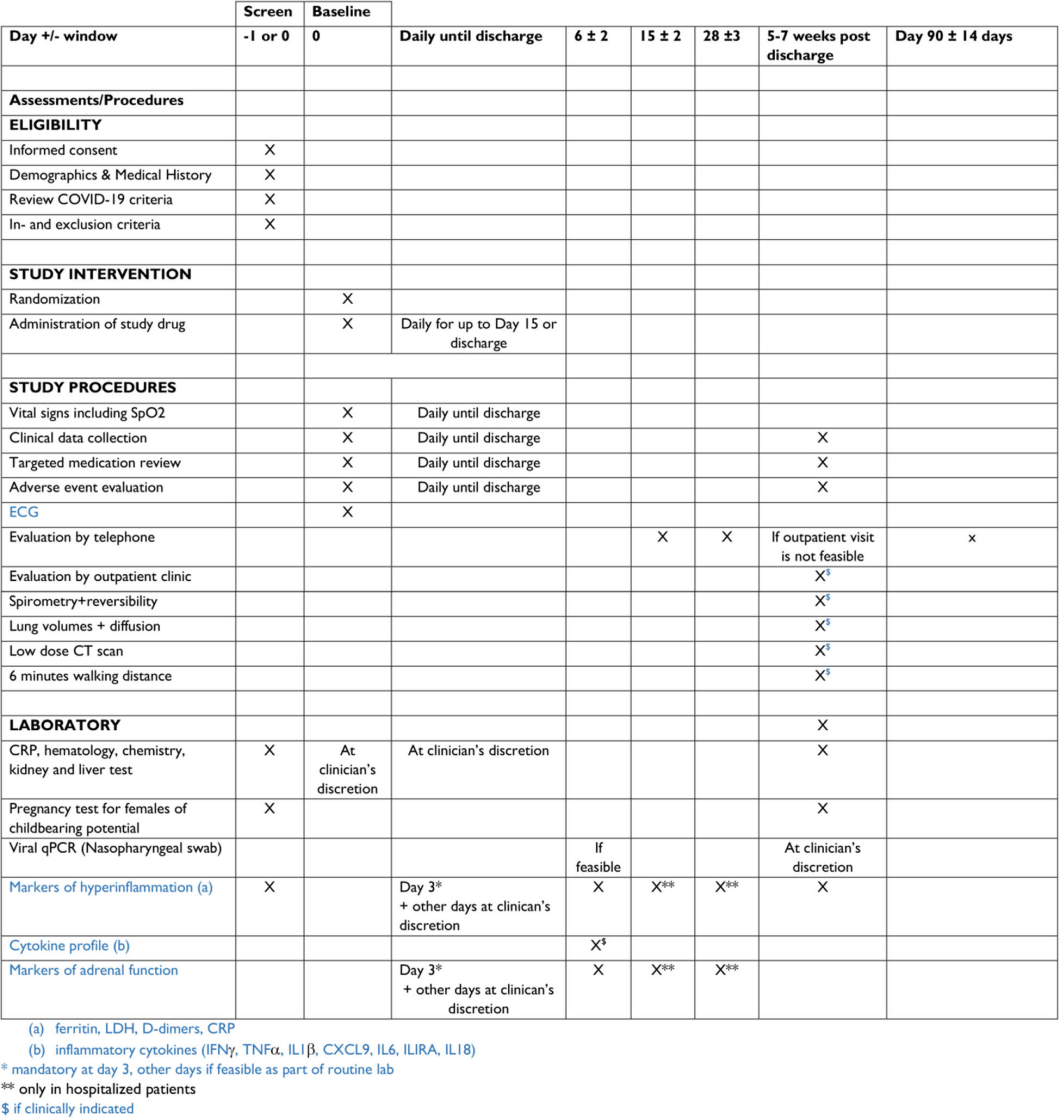
Participant timeline

### Sample size {14}

Sample size estimates provided serve as a reference, but not as an indication of the final number of patients to be randomized.

In the pilot phase of DAWn-AntiCo, we will evaluate the effect of modulation of the thromboinflammatory response on biochemical parameters. To evaluate the effect of this strategy, we will evaluate the difference in mean D-dimers on day 6. Published data show highly variable D-dimer values in patients with COVID [2, 3]. For an estimated mean D-dimer of 1000 with a standard deviation of 600, a sample size of 51 patients (17:34 with 1:2 randomization) has a power of 0.8 and an alpha of 0.05 to detect a difference of 500. We will therefore evaluate the effect of the intervention after a pilot phase of 60 (20:40) patients. Based on the information from other DAWn studies, the final sample size for effect on clinical outcomes will be evaluated following this pilot phase.

In their study comparing clinical improvement rates for lopinavir-ritonavir in hospitalized patients with severe COVID-19, Cao et al. reported a clinical improvement rate in the control group of 37.7% on day 14 [25].

Therefore, for our sample size calculations, we assume that a 40% improvement rate will be observed on day 15 in the control group, which yielded an expected hazard ratio of 1.5632. Since all missing clinical status data up to day 15 will be imputed, we assumed no censoring of the data prior to day 15. Based on the log-rank test, with a 2-sided significance level of 5% and 80% statistical power and using a (2:1) randomization ratio in favor of the intervention, we estimate that a total sample size of 354 patients will suffice to detect an absolute improvement of 15% (i.e., 55% in the intervention group). To detect an absolute improvement of 20% (60% in the intervention group), a total sample of 196 patients will suffice. We propose a pragmatic sample size of 210 patients taking into account early dropouts.

### Recruitment {15}

All patients diagnosed with COVID-19 are screened at the emergency ward or upon arrival at the COVID-ward. Accordingly, an emergency physician or a supervising physician at the COVID-ward that is trained in the protocol will first assess the patient’s interest in the study, if necessary through the legal representative. If there is an interest in participating, the investigators of the DAWn-study team again check the eligibility criteria and contact the patient or legal representative to provide more information and obtain informed consent. Therefore, every hospitalized patient with COVID-19 is screened upon hospitalization.

## Assignment of interventions: allocation

### Sequence generation {16a}

A randomization procedure through a computerized system (Research Electronic Data Capture or REDCap) has been established, generated by the data management unit of the clinical trial center Leuven, which ensures the integrity of the trial.

### Concealment mechanism {16b}

In this open-label study, patients are randomly allocated in order of diagnosis using a computerized system (REDCap) applying an unknown allocation sequence.

### Implementation {16c}

The investigators will randomize the patients using a computerized system (REDCap).

## Assignment of interventions: blinding

### Who will be blinded {17a}

As it is an open-label study, the study participants and clinical staff are not blinded.

### Procedure for unblinding if needed {17b}

The clinical staff is not blinded.

## Data collection and management

### Plans for assessment and collection of outcomes {18a}

Trained investigators and CTAs perform data collection daily. All required patient data are available in the patient’s electronic health file. Access to this file and its components is centrally monitored, making sure only the consented data are collected. The clinical scale proposed by the WHO will be used to assess clinical outcomes. The local laboratory of participating sites performs laboratory tests—the central lab tests interleukins in UZ Leuven.

### Plans to promote participant retention and complete follow-up {18b}

An outpatient follow-up is provided after 5–7 weeks with functional, radiographic, and laboratory evaluation, as described above. However, if this is not possible or participants discontinue the study, a short follow-up over the telephone will be suggested alternatively.

### Data management {19}

Data are stored and pseudonymized in the REDCap system allowing for secure and GDPR-compliant data handling.

### Confidentiality {27}

The data are collected, pseudonymized, stored, and managed in the REDCap system. This system allows for secure data management. Access to pseudonymized patient data is logged and only available to those who need data access. Patient data are securely stored in this system for 25 years, keeping up with the high standards of data protection.

### Plans for collection, laboratory evaluation, and storage of biological specimens for genetic or molecular analysis in this trial/future use {33}

Routine laboratory tests will be performed as described above. Citrated plasma leftovers of the samples used for these laboratory tests will be stored in a study-specific area in the University Hospitals Biobank at − 80 °C for future analysis.

## Statistical methods

### Statistical methods for primary and secondary outcomes {20a}

#### Analysis of the primary efficacy endpoint

The primary endpoint will be analyzed using competing risk analyses whereby death without any improvement will be considered as a competing risk. Event rates will be estimated using cumulative incidence functions (CIF). Median times to improvement will be calculated by the treatment group.

The effect of treatment will be assessed by performing a Fine and Gray competing risk regression model that includes the baseline clinical status value on day 0 as a covariate and randomized treatment as a factor. From the Fine and Gray model, the treatment effect and associated 95% confidence interval will be estimated.

In the pilot phase of DAWn-Antico, the effect of modulation of thromboinflammation will be evaluated on a laboratory primary efficacy endpoint, mean D-dimer level at day 6. The null hypothesis being tested is that the mean D-dimer level on day 6 is the same for the standard of care and intervention arm. Means will be compared with appropriate statistical testing after checking for normality. The full statistical analysis plan will be finalized before the database lock.

To evaluate the effect of aprotinin and high-dose enoxaparin vs. anakinra, analyses will be performed separately in patients with or without signs of hyperinflammation (i.e., with or without an indication for anakinra in the intervention group).

#### Analysis of the secondary endpoint(s)

1. Cumulative clinical status up to day 15 will be analyzed using a general linear model adjusted for clinical status on day 0. The treatment effect will be estimated by the difference of mean values between the groups.
2. Cumulative clinical status recorded daily during the hospital stay and on days 15 and 19 will be analyzed using a proportional odds logistic regression model, adjusted for clinical status on day 0. The common odds ratio will estimate the treatment effect.
3. All-cause mortality rates will be estimated by the treatment group using the Kaplan-Meier method. The resulting Kaplan-Meier curves will be compared using a log-rank test. The treatment effect will be estimated by the hazard ratio using a Cox regression.
4. Time-to-event parameters with competing risk (time to clinical improvement, composite cardiac endpoint): event rates will be estimated using cumulative incidence functions (CIF), and the resulting CIF curves will be compared using Gray’s test. The subdistribution hazard ratio will estimate the treatment effect.
5. Duration of hospital and ICU stay: both parameters will be analyzed as time-to-event parameters with competing risk, whereby the event of interest is discharged from hospital/ICU, and the competing risk is hospital/ICU death.
6. Continuous normally distributed variables (e.g., QTc) will be analyzed using a 2-sample *t* test. Treatment effects will be estimated by the difference in mean values between the groups. If applicable, changes from baseline will be calculated. Comparisons between treatment groups will be made by performing an analysis of covariance (ANCOVA) on the post-baseline value, using the baseline value as a covariate.
7. Continuous non-normally distributed variables (clinical status, NEWS score, duration of supplemental oxygen, duration of mechanical ventilation) will be analyzed using a Wilcoxon rank-sum test. Change in ordinal scale at specific time points will be compared using Wilcoxon rank-sum tests.
8. The proportion of patients with D-dimer < 1000, ferritin < 1000, and LDH < 600 on day 6 and day 15 will be compared.
9. The proportion of patients who develop hyperinflammation at days 3, 6, and 15; time to hyperinflammation.

### Interim analyses {21b}

An independent data monitoring committee will monitor the interim trial results. If at any stage evidence emerges that any one treatment arm/stratum is inferior, then it can be decided that that arm/stratum will be discontinued. Conversely, if good evidence emerges while the trial is continuing that some other treatment(s) should also be being evaluated, then it can be decided that one or more extra arms or strata will be added while the trial is in progress.

### Methods for additional analyses (e.g., subgroup analyses) {20b}

To evaluate the effect of aprotinin and high-dose enoxaparin vs. anakinra, analyses will be performed separately in patients with or without signs of hyperinflammation at randomization (i.e., with or without an indication for anakinra in the intervention group).

### Methods in analysis to handle protocol non-adherence and any statistical methods to handle missing data {20c}

Patients who do not have complete clinical status data up to day 15 will be accounted for as follows:

- Single-day missing, with preceding and following day known: in these cases, the missing value will be imputed by the maximum of the two surrounding values.
- Otherwise: missing data will be imputed using multiple imputation methodology whereby a total of 100 imputations will be done. Clinical status on each day will be imputed consecutively based on the clinical status of the previous days and meaningful clinical variables. When all data have been imputed, the cumulative score up to day 15 will be calculated using the imputed scores. Age, gender, baseline disease severity, and randomized treatment group will be included in the imputation model.

Up to the time of death, missing D-dimers will be imputed using a methodology similar to the one described for the imputation of clinical status. If necessary, a log-transformation will be applied to the D-dimer data to attain a normal distribution for D-dimer. After imputation, to account for deaths before day 6, D-dimer data will be analyzed using a GEE model that evaluates the data over time with an appropriate model.

### Plans to give access to the full protocol, participant-level data, and statistical code {31c}

The full protocol is available as an appendix to this paper. At this time, there is no public patient dataset planned. The datasets analyzed during the current study are available from the corresponding author on reasonable request.

## Oversight and monitoring

### Composition of the coordinating center and trial steering committee {5d}

A central multidisciplinary steering committee oversees the DAWn in the coordinating center, involved in strategic decisions and coordination between the different parallel studies. The steering committee members have complementary clinical interests such as infectiology, pulmonology, coagulation and bleeding disorders, and biomedical statistics. Specific DAWn-Antico tasks are delegated to a project management group, also in the coordinating center. The project management group consists of a project leader, the primary investigator and co-investigators, and dedicated study personnel. It supervises the recruitment and trial progress and is immediately informed when AEs occur to ensure proper communication to the other participating centers. The co-investigators of different studies of the DAWn consortium together operate a central command post, to ensure 24/7 availability for troubleshooting and supervising study-related procedures.

### Composition of the data monitoring committee, its role and reporting structure {21a}

Monitoring of the trial will be performed by qualified individuals (independent from the site trial staff) according to the monitoring plan. The sponsor and investigator/participating site will permit direct access to the trial data and corresponding source data and any other trial-related documents or materials to verify the accuracy and completeness of the data collected.

The data safety and monitoring committee (DMC) consists of 6 qualified members, independent from the site trial staff. Their scientific independence is assured through a DMC charter and terms of reference. Because of the exceptional circumstances, the DMC is part of UZ Leuven.

DMC will monitor ongoing results to ensure patient well-being and safety as well as study integrity. It will be asked to recommend early termination or modification only when there is clear and substantial evidence of a safety issue.

### Adverse event reporting and harms {22}

Investigators will seek information on AEs during each patient contact. All events, whether reported by the patient or noted by trial staff, will be recorded in the patient’s medical record and the electronic case report form or (e)CRF within a reasonable time after becoming aware. If available, the diagnosis should be reported on the AE form, rather than the individual signs or symptoms. If no diagnosis is available, the investigator should record each sign and symptom as individual AEs.

### Frequency and plans for auditing trial conduct {23}

The investigator will permit direct access to trial data and documents for monitoring, audits, and/or inspections by authorized entities such as but not limited to the following: the sponsor or its designees and competent regulatory or health authorities. As such, eCRFs, source records, and other trial-related documentation (e.g., the Trial Master File, pharmacy records) must be kept current, complete, and accurate at all times.

### Plans for communicating important protocol amendments to relevant parties (e.g., trial participants, ethical committees) {25}

As per good practice, trial participants will be informed of significant changes during the trial. Yearly updates will be given to the ethical committee.

### Dissemination plans {31a}

The primary paper will be published in a peer-reviewed journal and presented at international meetings.

## Discussion

The global scientific community is currently searching around the clock for treatments that can modify the disease course of COVID-19. Most of these trials focus on drugs that suppress viral replication or that provide passive immunization (convalescent plasma). Our approach provides an additional and complementary approach for patients with COVID-19 with an intervention that targets the inflammatory and thrombotic activation triggered by SARS-CoV-2. Several reports have already emphasized that the excessive thromboinflammatory response represents one of the most significant adverse prognostic factors in patients infected with SARS-CoV-2. Therefore, there is a clear rationale for testing drugs, explicitly targeting this response on multiple levels.

In this trial, we will test higher anticoagulant doses for thromboprophylaxis in combination with aprotinin. Aprotinin is a potent kallikrein inhibitor, and preliminary experiments in our center have shown that the addition of aprotinin to BAL fluid of COVID-19 patients completely abolishes kallikrein activity. It is a very attractive therapeutic option because it could potentially have a beneficial effect on both the viral cellular entry [7] and the thromboinflammatory response in COVID-19. The potentially beneficial role of aprotinin in treating acute lung injury provoked by SARS-CoV-2 has also recently been reviewed by Solun et al. [26]. In the case of hyperinflammation, anakinra will be added to the therapy. Other therapeutic options targeting hyperinflammation, such as IL-6 blockade, are also being tested in COVID-19. In this trial, we have chosen to block IL-1 with anakinra because of promising results in case series with COVID-19 [17] and a retrospective cohort study in which treatment with anakinra was safe and associated with clinical improvement in a majority of patients [18].

This study has several limitations. Firstly, the adaptive design that allows changes to the standard of care treatment depending on the latest evidence in a rapidly evolving scientific field might pose a challenge to the interpretation of the results. Secondly, the highly unpredictable epidemiology of the pandemic makes it challenging to predict if we will reach the targeted patient numbers in due time. While the study started during the Belgian peak, the incidence rapidly decreased after rigorous quarantine measures taken by the Belgian government. Similar trials are being conducted elsewhere, and results of those could urge early termination of our study, on the condition that similar dosing regimens have been used.

Despite the possible pitfalls, the study also has different strengths. Firstly, our trial consists of a unique approach based on the presumed pathophysiology of COVID-19. Secondly, the flexible trial design allows us to adapt the intervention arm based on new scientific insights. Finally, by using the WHO scale, our study results can and will be merged with other results of ongoing international trials to provide strong type A. Taken together, this study will identify the effect of a combination of LMWH with aprotinin and anakinra on the thromboinflammatory response and clinical outcome of COVID-19 patients.

## Trial status

The protocol attached (version 2.1) dates from 12 May 2020. Recruitment began on 20 May with the first patient being enrolled on 25 June. Further recruitment mainly depends on the event rate of the COVID-19 pandemic.

## Supplementary Information

**Additional file 1.**

**Additional file 2.**

## Abbreviations

ACE2: Angiotensin-converting enzyme 2
AE: Adverse event
ANCOVA: Analysis of covariance
aPTT: Activated partial thromboplastin time
CIF: Cumulative incidence functions
CrCl: Creatinine clearance
COVID-19: Coronavirus infectious disease-19
CTA: Clinical trial assistant
DMC: Data safety and monitoring committee
ECMO: Extracorporeal membrane oxygenation
(e)CRF: Electronic case report form
ICH: International Conference on Harmonization of Technical Requirements for Registration of Pharmaceuticals for Human Use
ICU: Intensive care unit
GCP: Good Clinical Practice
GI: Gastrointestinal
IV: Intravenous
LMWH: Low molecular weight heparin
MAR: Missing at random
MCAR: Missing completely at random
REDCap: Research Electronic Data Capture
SAE: Serious adverse event
SARS-CoV-2: Severe acute respiratory syndrome coronavirus 2
SOP: Standard operating procedure
