# Correction to: A randomized, open-label, adaptive, proof-of-concept clinical trial of modulation of host thromboinflammatory response in patients with COVID-19: the DAWn-Antico study

**DOI:** 10.1186/s13063-020-04991-y

**Published:** 2020-12-29

**Authors:** T. Vanassche, M. M. Engelen, Q. Van Thillo, J. Wauters, J. Gunst, C. Wouters, C. Vandenbriele, S. Rex, L. Liesenborghs, A. Wilmer, P. Meersseman, G. Van den Berghe, D. Dauwe, G. Verbeke, M. Thomeer, T. Fivez, D. Mesotten, D. Ruttens, L. Heytens, I. Dapper, S. Tuyls, B. De Tavernier, P. Verhamme, DAWn consortium members

**Affiliations:** 1 Center for Molecular and Vascular Biology, KU Leuven Department of Cardiovascular Sciences, KU Leuven, Leuven, Belgium; 2 Department of Cardiovascular Sciences, University Hospitals Leuven, Leuven, Belgium; 3 Center for Cancer Biology, VIB, Leuven, Belgium; 4 Department of General Internal Medicine, Medical Intensive Care Unit, University Hospitals Leuven, Leuven, Belgium; 5 Clinical Department and Laboratory of Intensive Care Medicine, Department of Cellular and Molecular Medicine, KU Leuven, Leuven, Belgium; 6 Pediatric Rheumatology, University Hospitals Leuven, Leuven, Belgium; 7 Laboratory of Adaptive Immunology & Immunobiology, Department of Microbiology and Immunology, KU Leuven, Leuven, Belgium; 8 Department of Anesthesiology, University Hospitals Leuven, Leuven, Belgium; 9 REGA Institute, KU Leuven, Leuven, Belgium; 10 Interuniversity Institute for Biostatistics and statistical Bioinformatics (I-BioStat), KU Leuven, Leuven, and Hasselt University (UHasselt), Hasselt, Belgium; 11 Department of Respiratory Medicine, Ziekenhuis Oost-Limburg, Genk, Belgium; 12 Department of Medicine and Life Sciences, Hasselt University, Diepenbeek, Belgium; 13 Department of Anaesthesiology, Intensive Care, Emergency Medicine and Pain Therapy, Ziekenhuis Oost-Limburg, Genk, Belgium; 14 Department of Anesthestiology, GZA hospital group, Antwerp, Belgium; 15 Emergency Medicine and Intensive Care, GZA hospital group, Antwerp, Belgium; 16 Respiratory Medicine, GZA hospital group, Antwerp, Belgium

**Correction to: Trials (2020) 21:1005**

**https://doi.org/10.1186/s13063-020-04878-y**

Following publication of the original article [1], we were notified that the figure of the study design in the paper was an older, incorrect figure that did not correctly reflect randomization strategy, patient numbers, and intervention. The incorrect and correct figures are presented below.

- Originally published figure 
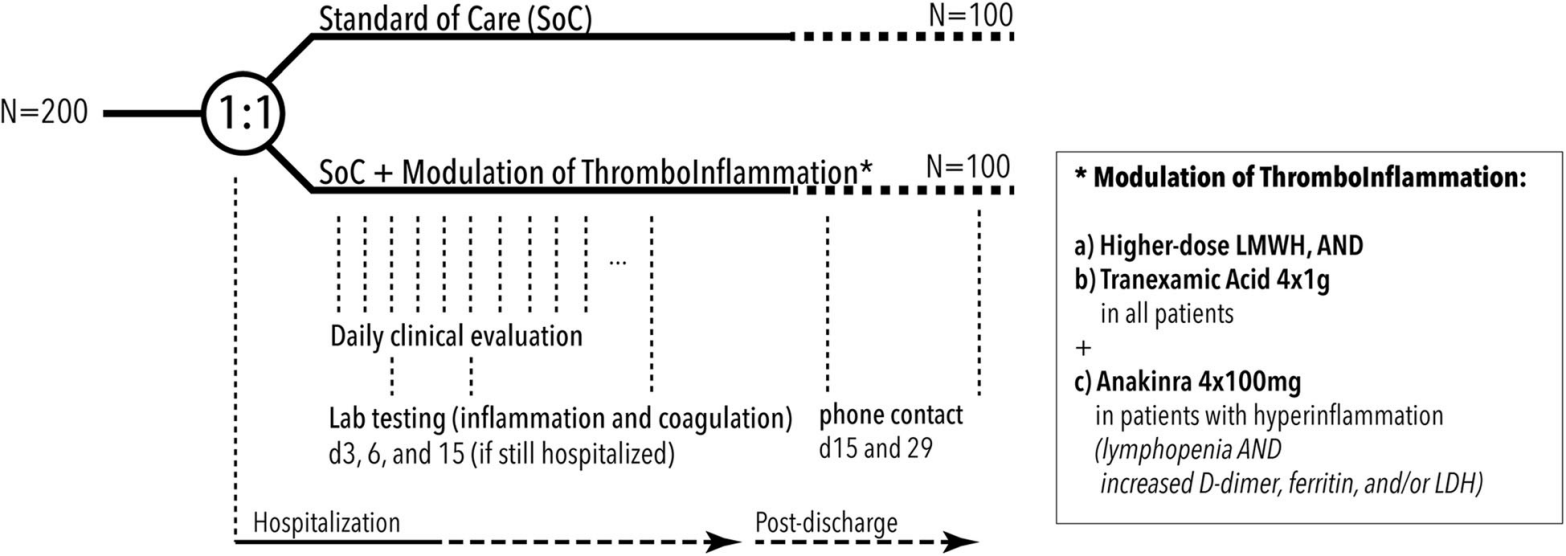
- Corrected figure 
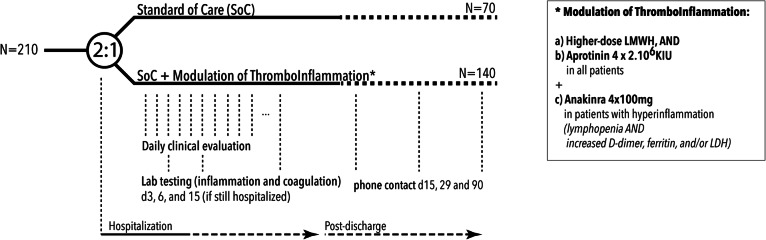

The original article has been corrected.
