# Supplementary material for: A randomized, open-label, adaptive, proof-of-concept clinical trial of modulation of host thromboinflammatory response in patients with COVID-19: the DAWn-Antico study
Source: Trials. 2020 Dec 9;21:1005. doi: 10.1186/s13063-020-04878-y (PMC7724460; doi:10.1186/s13063-020-04878-y)
Supplement: Supplementary file 1 — Additional file 1. [file 13063_2020_4878_MOESM1_ESM.pdf]

CLINICAL TRIAL PROTOCOL

A randomized, open-label, adaptive, proof-of-concept clinical trial of modulation of host thromboinflammatory response in patients with COVID-19.

Part of Direct antivirals working against nCoV (DAWN) studies

DAWN ANTICO v2.1 – Date May 05th, 2020

EudraCT Nbr: 2020-001739-28

Sponsor

University Hospitals Leuven (UZ Leuven)

Herestraat 49, B-3000 Leuven

Coordinating investigators

DAWN ANTICO – Prof Dr Thomas Vanassche

Part of DAWN study program

Coordination DAWN program - Prof Dr Peter Verhamme

Confidentiality Statement

The information in this document is strictly confidential and is available for review to Investigators, potential Investigators and appropriate Ethics Committees, Institutional Review Boards or Competent Authorities. No disclosure should take place without written authorization from the Sponsor.

CLINICAL TRIAL PROTOCOL HISTORY

CTP / Amendment #	Date	Reason for amendment
CTP/ DAWN AntiCo v1	09/04/2020	n/a
CTP/DAWN AntiCo v2.0	24/04/2020	Implementation comments EC/FAGG<br>Change in study intervention: switch from tranexamic acid to aprotinin<br>Change in participating centers (No participation of Virga Jessa Hasselt)
CTP/DAWN AntiCo v2.1	05/05/2020	Implementation comments EC/FAGG on protocol version 2.0

LIST OF PARTICIPATING SITES

(as applicable)

List Of Participating Sites	Principal Investigator
**DAWN**	
**DAWN AntiCo**	
UZLeuven	Prof. Dr. Thomas Vanassche<br>Prof. Dr. Joost Wauters<br>Prof. Dr. Jan Gunst<br>Prof. Dr. Carine Wouters<br>Dr. Christophe Vandenbriele<br>Prof. Dr. Steffen Rex
ZOL, Genk	Dr. Michiel Thomeer<br>Dr. Tom Fivez<br>Dr. Dieter Mesotten<br>Dr. David Ruttens
GZA	Dr. Luc Heytens

SIGNATURES

Title: A randomized, open-label, adaptive, proof-of-concept clinical trial of modulation of host thromboinflammatory response in patients with COVID-19.

Protocol: Part of Direct antivirals working against nCoV (DAWN) studies

The undersigned confirms that the above-referenced protocol has been acknowledged and accepted, and agree to conduct the Trial in compliance with the approved protocol, and will adhere to the principles outlined in the requirements for the conduct of clinical trials in the EU as provided for in Directive 2001/20/EC or the EU Regulation 536/2014 (as soon as in effect) and any subsequent amendments thereto, the ICH guidelines, the most recent version of the Declaration of Helsinki, the Belgian law of May 7th, 2004 regarding experiments on the human person (as amended) or the Belgian law of May 7th, 2017 related to clinical trials on medicinal products for human use (as soon as in effect), the EU General Data Protection Regulation 2016/679 (GDPR), the relevant Belgian laws implementing the GDPR, the Belgian Law of August 22nd, 2002 on patient rights, the Sponsor's applicable SOPs, and other regulatory requirements as applicable.

The undersigned agrees not to disclose the confidential information contained in this document for any purpose other than the evaluation or conduct of the Trial, without the prior written consent of the Sponsor.

The undersigned also commit to making the findings of the Trial publicly available through publication and/or other dissemination tools, following this protocol and applicable regulations, without any unnecessary delay and to provide an honest, accurate and transparent account of the Trial; and to explain any discrepancies or deviations from the approved Trial protocol.

Coordination DAWN Study

Prof. Dr. Peter Verhamme	....	....
Name & Title	Signature	Date

Coordinating Investigator

DAWN ANTICO

Prof. Dr. Thomas Vanassche	....	....
Name & Title	Signature	Date

Principal Investigator (Participating Site) *(in case of monocentric Trial, the Principal Investigator is the same as the Coordinating Investigator)*

DAWN ANTICO

....	....	....
Name & Title	Signature	Date

TABLE OF CONTENTS

Contents

Contents 1
1. Background and Rationale 5
2. Trial Objectives and Design 10
1.1 Trial objectives 10
1.2 Trial outcomes 11
1.2.1 Primary outcome 12
1.2.2 Secondary outcomes 12
1.2.3 Exploratory long-term outcomes 13
1.3 Trial Design 13
1.4 Expected Duration of the Trial 14
1.5 End of trial definition 14
2 Trial Population / Eligibility Criteria 15
2.1 Inclusion criteria 15
2.2 Exclusion criteria 15
3 Trial Procedures 16
3.1 Participant Consent and withdrawal of consent 16
3.2 Selection of Participants / Recruitment 17
3.3 Randomization Procedure 17
3.4 Trial Procedures 17
3.4.1 By visit 17
3.4.2 Laboratory tests 18
3.4.3 Other investigations 18
3.4.4 Exploratory investigations 18
3.5 Premature discontinuation of Trial treatment 18
4 Trial Medication / Drug 19
4.1 Investigational Medicinal Product and Dosing Regimen 19
4.2 Concomitant / Prohibited Medication / Treatment 22
4.3 Use of rescue anti-inflammatory therapy 22
5 Safety 22
5.1 Specification, timing, and recording of safety parameters 22
5.1.1 Definitions 23
5.1.2 Adverse Events that do not require reporting 23

5.1.3 Recording and reporting of Adverse Events 24
5.1.4 Assessment 24
5.1.5 Timelines for reporting 24
5.1.6 Follow-up 25
5.1.7 Death 25
5.1.8 Reporting requirements to Ethics Committee's (EC's) and Competent Authorities (CA's) 25
5.1.9 Sponsor's reporting of Suspected Unexpected Serious Adverse Reactions (=SUSARs) 25
5.1.10 Annual reporting 26
5.1.11 Data and Safety Monitoring Board (DSMB) and Treatment stopping rules 26
5.1.12 Communication plan to report relevant safety findings to all stakeholders 26
6 Statistics and Data Analysis 28
The null hypothesis being tested is that the primary outcome is the same for the standard of care and experimental treatment arms. 29
6.1 Sample Size Determination 29
6.2 Statistical Analysis 29
6.2.1 Population for analysis 29
6.2.2 Statistical Analyses 30
Data and monitoring safety board (DSMB) 32
7 Data handling 33
7.1 Data Collection Tools and Source Document Identification 33
7.1.2 Legal requirements 33
7.2 Audits and Inspections 34
7.3 Monitoring 34
7.4 Archiving 34
8 Ethical and Regulatory Considerations 35
8.1 Ethics Committee (EC) review & reports 35
8.2 Regulatory Compliance 35
8.3 Protocol / GCP compliance 35
8.4 Data protection and participant confidentiality 35
8.5 Insurance 36
8.6 Amendments 36
8.7 Post-Trial activities 36
9 Research Registration, Dissemination of Results and Publication Policy 36
10 Intellectual Property 37
11 Joint Commission International (JCI) 37
12 References 38
13 Appendix 1: Data Processing Annex (DPA) 40

LIST OF ABBREVIATIONS

Abbreviation	Definition
(e)CRF	(electronic) Case Report Form
AE	Adverse Event
AESI	Adverse Event of Special Interest
APR	Annual Progress Report
ASR	Annual Safety Report
AR	Adverse Reaction
CA	Competent Authority
CI	Coordinating Investigator
CM	Concomitant Medication
CSR	Clinical Study Report
CTP	Clinical Trial Protocol
DMP	Data Management Plan
DPA	Data Processing Annex
DSMB	Data Safety Monitoring Board
DSUR	Development Safety Update Report
EC	Ethics Committee
ECG	Electrocardiogram
EoT	End of Trial
FPFV	First Patient First Visit
GCP	Good Clinical Practice (latest version of ICH E6)
IB	Investigator's Brochure
ICF	Informed Consent Form
ICH	International Conference on Harmonisation
IMP	Investigational Medicinal Product
ISF	Investigator Site File
LPLV	Last Patient Last Visit
MP	Monitoring Plan
PI	Principal Investigator (Participating Site)
PRO	Patient-Reported Outcome
SAE	Serious Adverse Event
SAP	Statistical Analysis Plan
SAR	Serious Adverse Reaction
SmPC	Summary of Product Characteristics
SUSAR	Suspected Unexpected Serious Adverse Reaction
TMF	Trial Master File
TSC	Trial Steering Committee

FUNDING AND SUPPORT

Funder	Type of Financial or Non-Financial Support
UZ Leuven	Financial and Non-Financial Support

No-fault liability insurance has been taken out by UZ Leuven for treating and/or compensating Trial participants who are harmed as a consequence of participation in the Trial.

ROLES AND RESPONSIBILITIES

The Principle Investigator (PI) is responsible for the conduct of the Trial at his/her Participating Site, and for protecting the rights, safety, and well-being of the Trial participants. As such, the PI must ensure adequate supervision of the Trial conduct at the Participating Site. If any tasks are delegated, the PI will maintain a log of appropriately qualified persons to whom he/she has delegated specified Trial-related duties. The PI will ensure that adequate training is provided and documented for all Trial staff before conducting assigned Trial-related activities.

It is the Coordinating Investigator's (CI's) responsibility to supervise the general conduct (e.g., Trial progress, communication, protocol training and support of the participating sites, annual reporting to the Ethics Committee (EC), end of Trial notification(s) and results reporting…) of the Trial. The CI fulfills both Investigator and Sponsor responsibilities, as outlined in the International Conference on Harmonisation – Good Clinical Practice (ICH-GCP) E6(R2) and applicable regulations.

PI and CI shall each be referred to as « Investigator(s)».

TRIAL SYNOPSIS

Title of clinical Trial («Trial»)	A randomized, open-label, adaptive, proof-of-concept clinical trial of modulation of host thromboinflammatory response in patients with COVID-19.
Protocol Short Title Acronym	**Part of Direct antivirals working against nCoV (DAWN) studies**
Trial Phase (I, II, III, IV)	phase II proof-of-concept study
Sponsor name	University Hospitals Leuven (UZ Leuven)
Coordinator DAWN Study	Peter Verhamme
Contact Address	University Hospitals Leuven – Internal Medicine
Contact Email	Peter.Verhamme@uzleuven.be
Contact Phone	+ 32 16 34 47 75
Medical condition or disease under investigation	COVID-19
Trial rationale	To evaluate clinical efficacy and safety of investigational therapeutics for COVID-19
Primary objective	The overall objective of the study is to evaluate the clinical efficacy and safety of different investigational therapeutics relative to the standard of care in patients hospitalized with COVID-19.
Secondary objective(s)	To evaluate the clinical efficacy of different investigational therapeutics as compared to one another or the control arm as assessed by Clinical Severity, Oxygenation, Ventilation, Hospitalisation.
Trial Design	Randomized, Open-label, Multicentre, Adaptive Study design
Outcomes	Clinical status of the subject until day 15 (on a 7-point ordinal scale):<br>1. Not hospitalized, no limitations on activities<br>2. Not hospitalized, limitation on activities;<br>3. Hospitalized, not requiring supplemental oxygen;<br>4. Hospitalized, requiring supplemental oxygen;<br>5. Hospitalized, on non-invasive ventilation or high flow oxygen devices;<br>6. Hospitalized, on invasive mechanical ventilation or ECMO;<br>7. Death.<br><br>Primary outcome will be time from Day 0 to sustained clinical improvement or life discharge, whichever comes first, whereby a sustained clinical improvement is defined as an improvement of ≥ 2 points vs the highest value of Day 0 and 1 and sustained for at least 3 days.<br><br>Exploratory secondary outcomes

**DAWN AntiCo**	
Contact Address CI	University Hospitals Leuven – xxx
Contact Email CI	Thomas.vanassche@uzleuven.be<br>DAWN-antico@uzleuven.be
Contact Phone CI	016340675
Trial permancy /emergency contact	Thomas Vanassche
Contact email	DAWN-antico@uzleuven.be
Contact phone	016346585
EudraCT number	2020-001739-28
Other public database nbr	/
Principal Investigators and Participating Sites	University Hospitals Leuven (UZ Leuven) — Dr. Thomas Vanassche<br>ZOL Genk — Dr. Michiel Thomeer<br>GZA — Dr. Heytens
Sample Size	Pilot phase of 50 patients followed by clinical phase for a total of 210 patients
IMP, dosage, and route of administration	A strategy of intensive modulation of thromboinflammation from day 0 to 15. This intervention consists of:<br>a) high-prophylactic dose of low-molecular weight heparin (LMWH:enoxaparin or fraxiparin) sc from day 0 to 15<br>b) Apronin 4x 2.10^6KIE iv from day 0 to 3 (total of 72 hours)<br>c) *in patients with biochemical evidence of hyperinflammation:* addition of Anakinra iv infusion 4x100mg/d<br><br>vs. standard thromboprophylaxis (prophylactic LMWH(enoxaparin or fraxiparin) as per local practice)<br><br>Other investigational products may be added as part of the adaptive study design
Active comparator product(s)	Standard thromboprophylaxis with low-molecular weight heparin as per local practice
Maximum duration of treatment and Follow Up of a Participant	15 days of treatment<br>90 days of follow-up
Maximum duration of the entire Trial	1 year
Date anticipated First Participant First Visit (FPFV)	30 April 2020
Date anticipated Last Patient Last Visit (LPLV)	unknown

TRIAL FLOWCHART DAWN-ANTICO

Schedule of Events – Trial specific Procedures / Assessments

	**Screen**	**Baseline**						
**Day +/- window**	**-1 or 0**	**0**	**Daily until discharge**	**6 ± 2**	**15 ± 2**	**28 ±3**	**5-7 weeks post discharge**	**Day 90 ± 14 days**
**Assessments/Procedures**								
**ELIGIBILITY**								
Informed consent	X							
Demographics & Medical History	X							
Review COVID-19 criteria	X							
In- and exclusion criteria	X							
**STUDY INTERVENTION**								
Randomization		X						
Administration of study drug		X	Daily for up to Day 15 or discharge					
**STUDY PROCEDURES**								
Vital signs including SpO2		X	Daily until discharge					
Clinical data collection		X	Daily until discharge				X	
Targeted medication review		X	Daily until discharge				X	
Adverse event evaluation		X	Daily until discharge				X	
ECG		X						
Evaluation by telephone					X	X	If outpatient visit is not feasible	x
Evaluation by outpatient clinic							X[$]	

Spirometry+reversibility							X[$]	
Lung volumes + diffusion							X[$]	
Low dose CT scan							X[$]	
6 minutes walking distance							X[$]	
**LABORATORY**							X	
CRP, hematology, chemistry, kidney and liver test	X	At clinician's discretion	At clinician's discretion				X	
Pregnancy test for females of childbearing potential	X						X	
Viral qPCR (Nasopharyngeal swab)				If feasible			At clinician's discretion	
Markers of hyperinflammation (a)	X		Day 3* + other days at clinican's discretion	X	X**	X**	X	
Cytokine profile (b)				X[$]				
Markers of adrenal function			Day 3* + other days at clinican's discretion	X	X**	X**		

(a) ferritin, LDH, D-dimers, CRP
(b) inflammatory cytokines (IFNγ, TNFα, IL1β, CXCL9, IL6, ILIRA, IL18)

* mandatory at day 3, other days if feasible as part of routine lab

** only in hospitalized patients

$ if clinically indicated

1. Background and Rationale

In December 2019, the Wuhan Municipal Health Committee identified an outbreak of viral pneumonia cases of unknown cause. Coronavirus RNA was quickly identified in some of these patients. This novel coronavirus has been designated SARS-CoV-2, and the disease caused by this virus has been designated COVID-19. Currently, there are no approved therapeutic agents available for coronaviruses[1].

The aim of the Direct antivirals working against nCoV (DAWN) study protocol is to investigate promising drug compounds in a proof-of-concept study. The design is adaptive, i.e., it allows to add and remove treatment arms and or strata for drug candidates based on the most updated information. The study complies with the recommendations for outcomes as outlined by the WHO master template protocol (https://www.who.int/emergencies/diseases/novel-coronavirus-2019/technical-guidance/early-investigations; and https://www.who.int/emergencies/diseases/novel-coronavirus-2019/global-research-on-novel-coronavirus-2019-ncov assessed on March 20th, 2020).

Based on the current information, two strategies hold promise for a successful reduction of COVID-19 disease burden. The first is, of course, the reduction of viral replication. In the Laboratory of Virology and Chemotherapy at the Rega Institute (KU Leuven), a library of existing drugs that were previously tested in clinical trials, of which some are available on the market, was screened for activity against SARS-CoV-2 with the aim to repurposing drugs for COVID-19. It is expected that several compounds will be identified that show antiviral activity in vitro, which subsequently need to be investigated in patients[2].

The second strategy is to modify the (in some cases, excessive) host response. Widespread systemic inflammation and subsequent activation of the coagulation and complement system have repeatedly been described in severe COVID-19[3-9]. Moreover, drugs influencing these pathways (e.g., anticoagulation) have suggested improved outcomes in some small and non-randomized observational studies[10,11]. New strata that may thus be added to the DAWN study include intensifying or adding anti-inflammatory molecules (e.g., interleukin receptor antagonists, or C1-esterase inhibitors).

DAWN-AntiCo: MODULATION OF HOST THROMBOINFLAMMATORY RESPONSE

In the majority of patients, the virus leads to self-limiting mild respiratory symptoms. However, up to 15% of patients develop severe respiratory symptoms characterized by hypoxic respiratory failure[1],requiring mechanical ventilation and associated with a high fatality. There are currently no approved therapeutic agents available for SARS-CoV-2. Understanding the pathophysiology and the search for effective treatments are currently a worldwide research priority. Accumulating clinical data shows that in SARS-CoV-2 infection, hyper-inflammation appears to be particularly prominent in patients with severe disease, as well as in non-survivors as compared to survivors[4,5,12,13]
Current understanding suggests that this thrombo-inflammatory response can be divided into at least two different cascades that are activated and that are interlinked.

Activation of kallikrein-bradykinin pathway
Internalization of SARS-CoV-2 involves binding to the cellular ACE2 receptor and the TMPRSS2 receptor, both of which are abundantly expressed in the respiratory epithelium[14]. Viral binding to ACE2 reduces ACE2 activity.
As ACE2 inactivates angiotensin I and II, a lack of ACE2 activity leads to overstimulation of the angiotensin-receptor type II (ATR)[15]. This results in a local inflammatory response, which leads to vasodilation and increased vascular permeability. ATR-activation also actives the kallikrein-bradykinin-pathway, which results in the production of bradykinins from high and low molecular-weight kininogen (HMWK and LMWK). ACE2 is also required for the degradation and neutralization of inflammatory bradykinins.

A loss of ACE2 function could lead to an overactivation of the kallikrein-bradykinin pathway, leading to pulmonary inflammation and edema. This results in local vasodilation and alveolar edema, which causes the typical clinical and radiological findings of bilateral diffuse infiltrates and ground-glass opacities, leading to hypoxic respiratory failure. Preliminary research at out center showed very high tissue kallikrein activity in broncho-alveolar fluid of COVID-19 patients, supporting this hypothesis.

Besides local inflammation, kallikrein-bradykinin system activation also results in the downstream activation of coagulation and of systemic inflammation.

Kallikreins link inflammation and coagulation
The HMWK-kallikrein-bradykinin system is also closely linked to the contact pathway of the coagulation system. HMWK and kallikrein activate clotting factor XII to fXIIa, and fXIIa, in turn, increases the activation of kallikrein from prekallikrein. Activation of the coagulation pathway leads to local fibrin deposition, as well as diffuse intravascular coagulation. Activation of the fibrinolytic system results in plasmin generation, which feeds back to increased factor XII and kallikrein activation. Interestingly, a more potent plasmin activity has been correlated with increased infectivity of SARS-CoV-2[9].
Patients with COVID-19 show signs of a strongly activated thrombotic pathway and hyperfibrinolysis. Increased D-dimers were found to be predictive of severe disease, and D-dimers > 1µg/mL was independently associated with an 18-fold higher risk of mortality in patients with COVID-19[1].

Cytokine storm activation
Asides from D-dimers, high ferritin, and high LDH levels were also found to predict poor outcome. Furthermore, initial data have shown the presence of hyperproduction of inflammatory cytokines with a profile similar to that present in patients with secondary hemophagocytic lymphohistiocytosis (sHLH)[13]. Additional unpublished data generated in the laboratory of Immunology of the National Institute for Infectious Diseases and the Laboratory of ImmunoRheumatology of the Ospedale Pediatrico Bambino Gesù confirm these findings with elevated levels of IL-1β, IL-6, TNF, and of the IFN-γ induced chemokines CXCL9 and CXCL10 being detected particularly in severe COVID-19 patients. Furthermore, these findings have been very recently confirmed in an analysis at University Hospitals Leuven (personal communication CW). Secondary HLH is most often associated with conditions in which chronic immune dysregulation occurs, such as in rheumatic diseases and certain hematologic malignancies, or triggered by infectious agents, particularly members of the herpes virus (Herpesviridae) family.
In terms of its pathologic origins, secondary HLH/MAS develops as part of a "cytokine storm". The proinflammatory cytokines that have been associated with secondary HLH/MAS include interferon-γ, interleukin-1 (IL-1), IL-6, IL-12, IL-18, and tumor necrosis factor[16,17].
The bradykinin-2 receptor links activation of the kallikrein-bradykinin system to systemic inflammation by triggering the interleukin-1 pathway. Furthermore, kallikrein also activates the complement system, further increasing the inflammatory response.

Proteases and cellular entry of SARS-CoV-2
In order to bind to ACE2, the spike protein of SARS-CoV-2 requires proteolytic modification that is essential for cellular entry and subsequent cytotoxicity and intracellular replication. The viral protease TMPRSS2 is required to prime the spike protein. A recent study has shown that inhibition of protease activity by aprotinin abolishes cellular infectivity and could prevent cytotoxic viral effects *in vitro*.

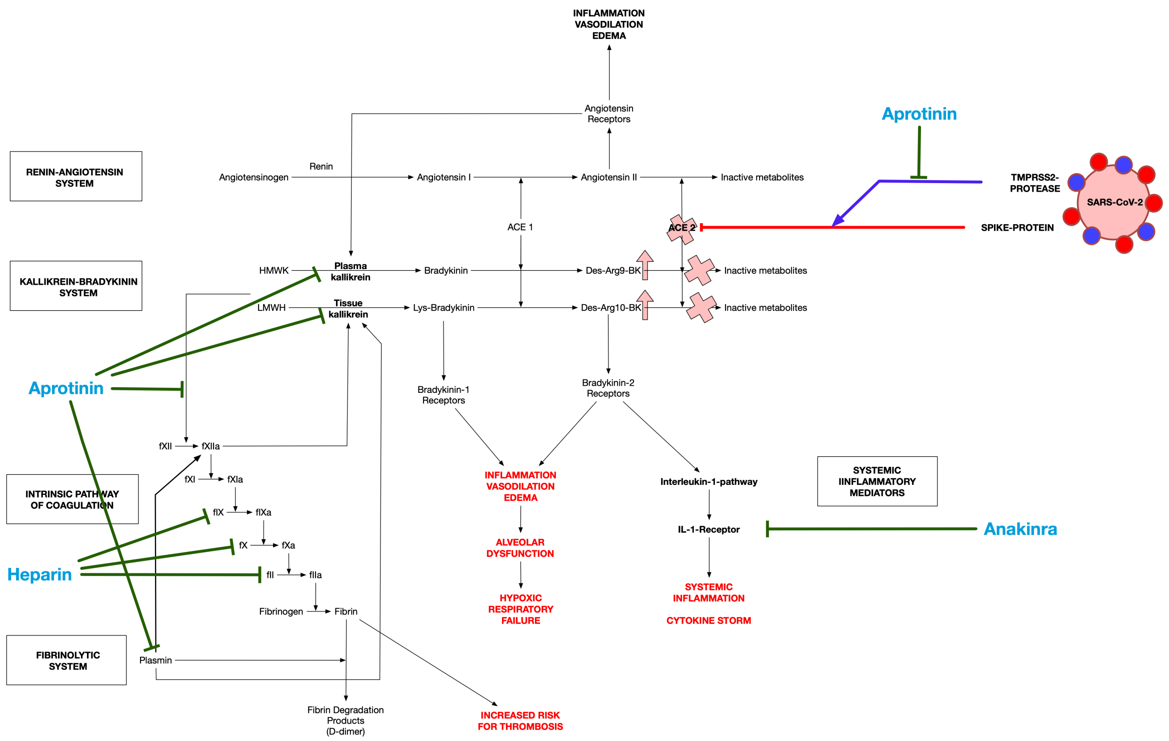

Modulating contact pathway activation and kallikrein-bradykinin pathway

The choice of kallikrein pathway inhibitor

Upstream inhibition of the activated bradykinin-kallikrein pathway could limit the pathophysiological changes triggered by SARS-CoV-2 infection and internalization and neutralization of ACE2. Several specific blockers of components of the contact pathway and the kallikrein-bradykinin pathway exist. However, none of those products is currently available for clinical use or has undergone safety testing. Recombinant C1-esterase-inhibitor can be used to supplement depleted counterregulatory mechanisms in patients with a genetic or acquired C1-esterase-inhibitor deficiency.

Tranexamic acid (TXA) binds reversibly to the lysine binding sites on plasminogen, thereby attenuating its capacity to bind to fibrin, which is essential for its activation by plasminogen activators. At higher concentrations, tranexamic acid also acts as an active site inhibitor of serine proteases, but is only a weak inhibitor (e.g. Ki of TXA for plasma kallikrein is approximately 30 mM). However, TXA is safe, cheap and widely available. Limited information is available in the literature about the relative inhibition by TXA of plasma vs tissue kallikrein. Therefore, we have performed **preliminary experiments to measure kallikrein activity** in the BAL fluid of patients with COVID-19 disease (experiments were part of the CONTAGIOUS trial, prof. Wauters Joost (co-investigator of this study) and prof. Wauters Els. (https://www.uzleuven.be/en/news/large-scale-leuven-research-coronavirus-and-covid-19).

These experiments highlighted that in the BAL fluid, mainly *tissue* kallikrein could be measured, whereas plasma kallikrein activity was absent or minimal in most patients. Furthermore, this experiments confirmed that TXA had only minimal or no effect on *tissue* kallikrein (unpublished confidential data, experiments were performed in collaboration with Johan Neyts, at the L3plus facility of the Rega Institute, and Marc Vanhove, Oxurion)

Aprotinin is a natural, globular polypeptide of 6512 Dalton, arranged in a single 58-amino acid chain cross-linked by three sulfide bridges and extracted from bovine lungs[18-2018-2018-2018-20]. It acts as a

competitive serine protease antagonist, **inhibiting various enzymes including but not limited to plasmin, tissue kallikrein, plasma kallikrein, tissue factor and PAR-receptors on platelets. Importantly, aprotinin also inhibits activation of factor XII by the HMWK-kallikrein pathway.** Aprotinin has been used to treat patients with pancreatitis, and is approved as an antifibrinolytic agent to prevent blood loss during cardiopulmonary bypass and cardiac surgery.

The Ki of aprotinin for plasma kallikrein is in the 30 nM range, for tissue kallikrein 20 nM range. Hence, aprotinin is more than 1 million fold more potent to inhibit plasma kallikrein, and has a Ki in the nanomolar range for tissue kallikrein[21,22]. In the BAL fluid of COVID-19 patients, addition of aprotinin completely abolished kallikrein activity *(unpublished data)*.

Potential antiviral effects of Aprotinin?

Potential antiviral effects of aprotinin against SARS-CoV-2 were highlighted in a recent studies.

(available as pre-published information:

Hoffmann, Kleine-Weber, Schroeder,et al. SARS-CoV-2 Cell Entry Depends on ACE2 and TMPRSS2 and Is Blocked by a Clinically Proven Protease Inhibitor. Cell 2020;

TMPRSS2 and furin are both essential for proteolytic activation 1 and spread of SARS2 CoV-2 in human airway epithelial cells and provide promising drug targets. Bestle et al.bioRxiv preprint doi: https://doi.org/10.1101/2020.04.15.042085.;

SARS-CoV-2 and SARS-CoV differ in their cell 1 tropism and drug 2 sensitivity profiles. Bojkova et al. bioRxiv preprint doi: https://doi.org/10.1101/2020.04.03.024257)

Cellular entry of SARS-CoV-2 requires priming of the spike-protein for binding to ACE2, which is dependent on the protease function of the viral capside protease TMPRSS2. Aprotinin acts as an inhibitor of this protease activity, resulting in a reduction of the cytotoxic effect of SARS-CoV-2 in cell culture. This provides an important additional rationale for selecting aprotinin as a combined inhibitor of viral cellular entry and subsequent cytotoxic effect and intracellular replication, and of the pathophysiological cascade that leads to local pulmonary inflammation as well as systemic hyperinflammation.

However, more detailed PK/PD studies and preclinical studies (e.g. in the COVID-19 precliical hamster model developed at the Rega-institutie in Leuven) are required before we can claim an antiviral effect of aprotinin. Hence, the rationale of this application for use of aprotinine entirely relies on its thrombo-inflammatory effect as a result of kallikrein inhibition.

Pharmacokinetic considerations for the use of aprotinin

The pharmacokinetic profile of aprotinin has been described previously[23,24]. In sum, aprotinin has been characterized by (at least) a biphasic patter, signified by a rapid distribution (half-life <1h) and then a slowed elimination (half-life <10h). Linear pharmacokinetics are suggested for doses ranging from 50000KIU to 2000000KIU [23]. When administered intravenously, it is readily distributed into tissues with about 10% of the activity reaching lung tissues [19]. Following a single intravenous dose of aprotinin, approximately 25-40% of the radioactivity is excreted in the urine over 48 hours. After a 2 million KIU dose infused over 30 minutes, urinary excretion of unchanged aprotinin accounted for approximately 9% of the dose. Aprotinin, after being filtered by the glomeruli, is actively reabsorbed by the proximal tubules in which it is stored in phagolysosomes. Aprotinin is then slowly degraded by lysosomal enzymes over multiple days .

Initial investigations targeted its use in hyperfybrinolytic conditions [23]. Its first clinical application was in 1953 in acute pancreatitis. Subsequent studies, from the 1960's and thereafter, mostly concerned (early) experiences in pancreatitis, shock and trauma. None of these investigations reached noteworthy positive results in terms of improved clinical outcome. A recent meta-analysis confirmed the neutral effects of aprotinin in pancreatitis [25]. In these investigations, an intermittent bolus regimen was commonly followed. In 1987 the Hammersmith group found a reduction of blood loss as an unexpected finding [26,27]. The Hammersmith group was among the first to systematically use a bolus/continuous infusion approach of aprotinin to reach a specific KIU/ml plasma target in order to maximally inhibit plasmin (and hence blood loss in a cardiac surgery setting).

In clinical practice, an intermittent bolus approach would however allow for easier aprotinin administration compared to a continuous infusion. Importantly, Clasen et al observed that a **single high dose infusion resulted in a sufficient high peak exposure** [28]. For the better part of a short infusion interval (e.g. 6 hours), sufficient trough levels can be reached using a sufficiently high bolus dose and **taking into account the target of tissue kallikrein inhibition, which is inhibited readily with plasma concentrations of <50KIU/ml** [28,29]. Hoffmann et al found that low concentrations, ranging from 4-10KIU/ml were already able to inhibit tissue kallikrein activity [30]. As sufficient activity has to still reach the lung tissues for the majority of time, a 2 MIE KIU bolus approach was selected. **A 2 MIE dose will achieve peak exposure in excess of 200 KIU/ml [29]. After 4-6 hours, it is expected that trough levels of about 40-50 KIU/ml will be achieved.** For DAWN-Antico the following regimen will hence be used: 2.10^6KIE bolus infusion over 30 min, q6h, for a total duration of 72 hours, totaling 8 MIE infused per 24h. A consensus was reached for this dosing regimen together with product specialists of Nordic Pharma and David Royston, who was paramount in elucidating aprotinin's use in cardiac surgery.

Trasylol is supplied by Nordic Pharma as a clear, colorless, sterile isotonic solution for intravenous administration with each milliliter containing 10,000 KIU (1.4 mg/mL). Trasylol will be administred in a separate iv line, no coadministration of other drugs is allowed over the same iv line.

Optimal thromboprophylaxis in patients with COVID-19

Heparins have a broad-spectrum anticoagulant effect. By potentiating antithrombin, low-molecular-weight heparins increase the endogenous inhibition of factors IX, X, and II by more than a thousand-fold. In a retrospective, non-controlled study, patients with COVID-19 who were treated with LMWH were more likely to survive than patients who did not receive anticoagulation[10,11]. The effect of LMHW on survival was more pronounced in patients with strongly elevated D-dimers. Standard doses of LMWH are routinely used for thromboprophylaxis in medically ill patients. However, recent guidance from Sciensano suggests that higher than usual prophylactic doses of LMWH could be considered, especially in the context of clinical trials. A recent study[31] showed a very high risk of venous thromboembolic complications in patients with severe COVID-19, despite usual doses of thromboprophylaxis.

Modulating cytokine storm

Anakinra is a recombinant form of the human IL-Ra, r-metHuIL-1Ra, which is produced by recombinant DNA technology in an E. coli expression system. Therapeutically, anakinra neutralizes the biological activity of IL-1 (IL-1α and IL-1β) by competitively inhibiting its binding to the IL-1RI.

The safety and benefits of IL-1 blockade in the inflammatory process have been demonstrated in many diseases, including in the treatment of systemic juvenile idiopathic arthritis (JIA) and systemic JIA–related MAS.

IL-1 receptor blockade was associated with significant improvement in survival of patients with sepsis and concurrent hepatobiliary dysfunction/disseminated intravascular coagulation and also in a series of patients with a clinical picture of secondary HLH/sepsis/multiple organ dysfunction syndrome/MAS that occurred in the setting of a variety of rheumatic and nonrheumatic diseases[32-34].

Taken together, hyper-inflammation, caused by a cytokine storm resulting from an exaggerated response of the immune system to the presence of the virus, is considered to represent one of the significant negative prognostic factors in patients infected with SARS-CoV-2, which constitutes the rationale for testing drugs specifically targeted to reduce the cytokine storm.

Kineret (anakinra) is a marketed drug first approved for the treatment of RA in the US in 2001 and subsequently in the EU/EEA in 2002. Kineret is also approved for the treatment of CAPS (in EU/EEA, Israel, and Australia), Still's disease, including SJIA and AOSD (in EU/EEA) and SJIA (in Australia).

An integrated intervention to target thromboinflammation in COVID-19

In sum, we hypothesize that an early suppression of the inflammatory pathways specifically upregulated as a result of SARS-CoV-2 infection, together with an inhibition of the downstream pathways of

hypercoagulability, hyperfibrinolysis, and interleukin-1 pathway, could reduce disease severity in patients with COVID-19.

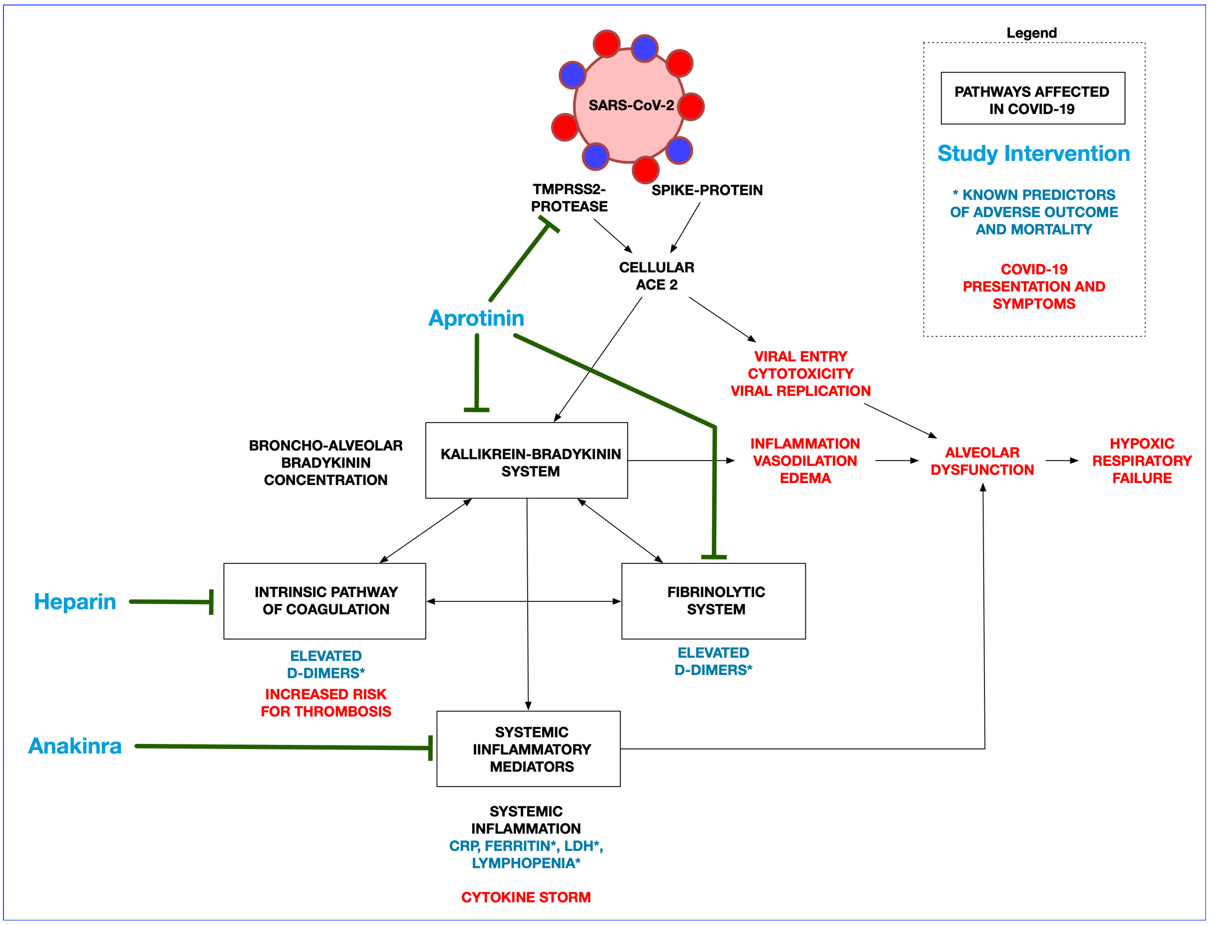

DAWN: overall

Eligible adult patients who tested positive for SARS-CoV-2 and are admitted to the hospital will be randomized and assessed daily during hospitalization. Discharged patients will be contacted by telephone at days 15, 29, and 90. All subjects will undergo efficacy and safety assessments, including laboratory assays, which are aligned with clinical care. Also, blood samples and nasopharyngeal swabs will be done according to clinical need (standard of care), and if feasible, an additional nasopharyngeal swab will be taken on day 6. The study should not put an extra burden on healthcare workers and the hospital's resources.

2. Trial Objectives and Design

1.1 Trial objectives

The study objectives are adapted from the WHO master protocol that was proposed to streamline interventional studies in patients with COVID-19. (https://www.who.int/emergencies/diseases/novel-coronavirus-2019/technical-guidance/early-investigations; https://www.who.int/emergencies/diseases/novel-coronavirus-2019/global-research-on-novel-coronavirus-2019-ncov assessed on March 20th, 2020).

The overall objective of the DAWN study is to evaluate the clinical efficacy and safety of investigational therapeutic agents relative to the standard of care in patients hospitalized with COVID-19. Additionally,

the effect of interventions targeting the host response modulation will be evaluated separately in a pilot phase by a primary laboratory endpoint (change in thrombo-inflammatory biomarkers), and clinical outcomes will be evaluated as a secondary outcome.
Secondary objectives are to evaluate the clinical efficacy of different investigational therapeutics as compared to one another or the control arm.

All DAWN studies will follow the same primary and secondary endpoints as assessed by:

Clinical Severity

Ordinal scale:

- Cumulative clinical status up to day 15
- Time from admission to an improvement of one category on an ordinal scale
- Subject clinical status on an ordinal scale at days 3, 5, 8, 11, 15, and 28.
- Mean change in the ranking on an ordinal scale from baseline to days 3, 5, 8, 11, 15, and 28 from baseline.

Host thrombo-inflammatory status (DAWN-AntiCo)

- D-dimer level on day 6, and D-dimer level on day 15 and 28
- CRP, ferritin, LDH level on day 6, and day 15 and 28
- Interleukin profile on day 6

Oxygenation

- Oxygenation free days in the first 28 days (to day 29).
- Incidence and duration of new oxygen use during the trial.

Mechanical Ventilation

- Ventilator free days in the first 28 days (to day 29).
- Incidence and duration of new mechanical ventilation use during the trial.
- Time to live weaning from mechanical ventilation

Hospitalization

- Duration of hospitalization (days).
- Time to live discharge from ICU
- Time to live discharge from hospital

Mortality

- 15-day mortality
- 28-day mortality
- 90-day mortality

Evaluate the safety of the interventions through 28 days of follow-up as compared to the control arm as assessed by

- Cumulative incidence of serious adverse events (SAEs) and adverse events (AEs) graded as severe.
- Discontinuation or temporary suspension of drug administration (for any reason).
- Changes in white cell count, haemoglobin, platelets, creatinine, glucose, total bilirubin, ALT, and AST over time.

1.2 Trial outcomes

The study outcomes are based on the WHO master protocol. All outcomes will be presented overall as well as separately for patients with mild/moderate vs severe disease at baseline.

1.2.1 Primary outcome

Pilot phase of DAWN-ANTICO:

D-dimer on day 6

Clinical status of subject at day 15 (on a 7-point ordinal scale):

1. Not hospitalized, no limitations on activities
2. Not hospitalized, limitation on activities;
3. Hospitalized, not requiring supplemental oxygen;
4. Hospitalized, requiring supplemental oxygen;
5. Hospitalized, on non-invasive ventilation or high flow oxygen devices;
6. Hospitalized, on invasive mechanical ventilation or ECMO;
7. Death.

Primary outcome will be time from Day 0 to sustained clinical improvement or life discharge, whichever comes first, whereby a sustained clinical improvement is defined as an improvement of $\geq$ 2 points vs the highest value of Day 0 and 1 and sustained for at least 3 days.

1.2.2 Secondary outcomes

- Status on an ordinal scale assessed daily while hospitalized and on days 15 and 28.
- Mortality on day 15 and day 28, time to death
- Time to clinical improvement (n° days from hospitalization to first 2-point improvement from highest previously recorded clinical state on the 7-point ordinal scale)
- Duration of supplemental oxygen.
- Duration of mechanical ventilation, time to live weaning from ventilation.
- Duration of hospitalization, time to live hospital discharge
- Duration of intensive care stay, time to live discharge from ICU
- Date and cause of death (if applicable).
- Use and doses of rescue anti-inflammatory therapy

- Adverse events graded as severe or SAEs, SARS, SUSARs.

- Lab values: including but not limited to CRP, white cell count, absolute neutrophil count, absolute lymphocyte count, absolute eosinophil count, hemoglobin, platelets, serum creatinine, eGFR (CKD-EPI) and CrCl (Cockroft-Gault), hsTroponin T, glucose, potassium, total bilirubin, ALT, and AST on days 1; 3, 5, 8, 11, 15 and 28 (If measured according to clinical indication).

- Markers of hyper-inflammation: (CRP, ferritin, LDH, interleukin profile) at baseline and on day 3, 6 and 15

- Markers of thrombotic activation and kallikrein-bradykinin activation: (D-dimers, fibrinogen, PT, aPTT, C1-inhibitor, factor XII) at baseline and on 3, 6 and 15

- Markers of adrenal function (Cortisol, ACTH, albumin/transcortin) at baseline and on day 3, 6 and 15

- Combined cardiac endpoint during hospitalization (any of the following: high sensitive troponin T levels >0.5ng/mL, ventricular arrhythmia requiring intervention, reanimation, sudden cardiac death)

- Incidence of thrombotic events during hospitalization

- Incidence of major bleeding complications during hospitalization as per ISTH criteria.
  ISTH major bleeding is defined as having a symptomatic presentation and
  - Fatal bleeding, and/or
  - Bleeding in a critical area or organ, such as intracranial, intraspinal, intraocular, retroperitoneal, intra-articular or pericardial, or intramuscular with compartment syndrome, and/or
  - Bleeding causing a fall in hemoglobin level of 20 g L^{-1} (1.24 mmol L^{-1}) or more, or leading to transfusion of two or more units of whole blood or red cells.
- Incidence of and time to hyperinflammation during hospitalization in both arms (i.e., meeting the criteria to receive anakinra if patient would be randomized in the intervention arm)
- Assess the effect of anakinra on CXCL9, IL-1, IL-6, IL18, TNF and selected biomarkers relevant for hyperinflammation, at day 6 and 15
- Assess the effect of thromboinflammatory modulation on D-dimer levels on day 6 and the highest D-dimer level during follow-up.

1.2.3 Exploratory long-term outcomes

- Qualitative and quantitative PCR for SARS-CoV-2 in (nasopharyngeal) swab on day 6 (when feasible in clinical care)

OVERALL: long-term exploratory outcome

- In patients who are invited for follow-up clinical evaluation 5-7 weeks post-discharge as per routine clinicnal indication:
  - Spirometry with reversibility
  - Lung volumes and diffusing capacity
  - Low dose CT scan
  - Laboratory
  - 6 minutes walk (at physicians discretion)
  - Transthoracic Echocardiography (TTE)

- A telephone call on D90±2 weeks post-admission for survival status

1.3 Trial Design

This **DAWN** study is an **adaptive, randomized, open-label clinical trial** to evaluate the safety and efficacy of promising antiviral agents in hospitalized adult patients diagnosed with COVID-19.
The outcomes of the study protocol are in part based on the draft master protocol of the WHO for trials that evaluate the safety and efficacy of investigational therapeutics for the treatment of COVID-19 in hospitalized patients. The study is a **phase 2 proof-of-concept multicenter trial program** consisting of various **interventions in patients with COVID-19**.

The DAWN study will compare the standard of care vs. standard of care with the investigational therapeutic agent or strategy. Since there are no current approved treatment options for COVID-19, the standard of care is mostly supportive. However, the standard of care will reflect the guidance by (inter)national guidelines and hence may change during the study. The clinical outcomes of this study have been chosen based on the outcomes of the WHO master template for clinical studies to allow pooling of the data with other ongoing studies.

The adaptive study design of DAWN allows for the addition of new treatment and strata during the study.

The **DAWN ANTICO** is a multi-center trial with 2:1 randomization evaluate standard thromboprophylaxis (low-molecular weight heparin at 50IU/kg once daily in hospitalized patients and twice daily in patients on intensive care units or as per local practice) with a multi-step antithrombotic and anti-inflammatory strategy. This strategy consists of

a) Higher prophylactic dose of LMWH: 50 IU/kg twice daily in hospitalized patients and 75IU/kg twice daily in patients on intensive care units from day 0 to 15, AND
b) Aprotinin $4x2x10^6$ KIE iv per day from day 0 to 3 for a total of 72 hours (3x4 doses)
c) Additionally, **patients with biochemical signs of hyperinflammation** at baseline or during follow-up will receive add-on interleukin-1-receptor blockage by Anakinra 100mg 4x daily

DAWN-AntiCo will start with a pilot phase powered to detect the biochemical response and will continue to detect the effect on clinical outcomes.

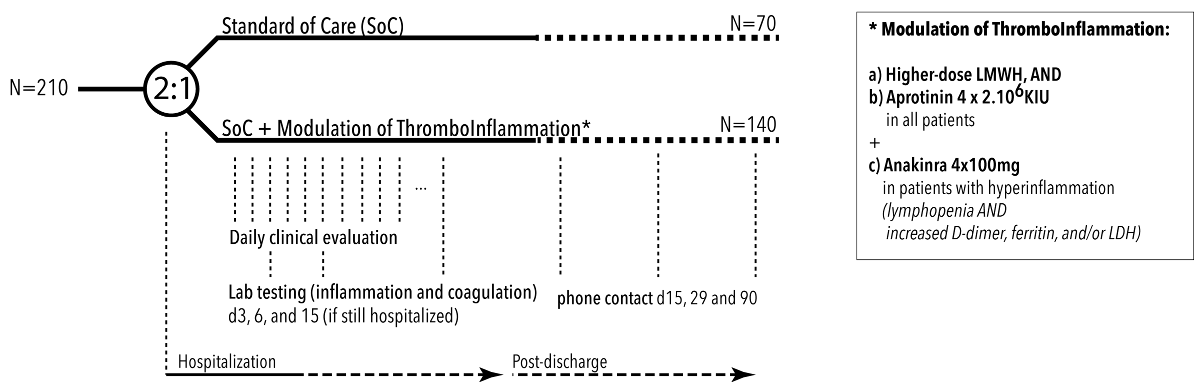

1.4 Expected Duration of the Trial

The **DAWN AntiCo** trial is expected to start in April 2020 with a duration of 1 year.

1.5 End of trial definition

End of trial is defined as the date of the last visit of the last patient in the trial.

2 Trial Population / Eligibility Criteria

2.1 Inclusion criteria

Participants eligible for inclusion in this Trial must meet **all** of the following criteria:

1. Subject (≥18 years old) or legally authorized representative provides informed consent prior to initiation of any study procedures. When signed informed consent is not possible (e.g. due to restrictions to prevent viral transmission), verbal informed consent in the presence of a witness will be obtained and documented in the medical files. Signed informed consent will be obtained as soon as the safety concerns are mitigated.
2. Subject (or legally authorized representative) understands and agrees to comply with planned study procedures.
3. Male or non-pregnant female adult ≥18 years of age at time of enrolment.
4. Has a **confirmed diagnosis of SARS-CoV-2 infection**, defined as *either:*
   a. laboratory-confirmed SARS-CoV-2 infection as determined by PCR, or other validated commercial or public health assay in any specimen as diagnosed within 72 hours prior to randomization
   or
   b. The combination of upper or lower respiratory infection symptoms (fever, cough, dyspnea, desaturation) **and** typical findings on chest CT scan **and** absence of other plausible diagnoses.
   In patients without PCR-confirmed diagnosis at inclusion, all efforts will be made to confirm definite SARS-CoV-2 infection, such as by PCR on bronchial aspirate or BAL fluid or by serologic testing. Participants who - despite all efforts – do not have a confirmed diagnosis of COVID-19 will be excluded from the analysis.

5. Illness of any duration, and at least one of the following:
   a. Radiographic infiltrates by imaging (chest x-ray, CT scan, etc.), or
   b. Clinical assessment (evidence of rales/crackles on lung auscultation) AND SpO2 ≤ 94% on room air, or
   c. Requiring mechanical ventilation and/or supplemental oxygen.

All participants that are considered for Trial participation, per the above criteria will be documented on the Screening Log, including Screen Failures.

2.2 Exclusion criteria

Participants eligible for this Trial must **<u>not</u>** meet any of the following criteria:

1. ALT/AST > 8 times the upper limit of normal.
2. Pregnancy or breastfeeding.
3. Allergy to any study medication.
4. Any medical condition which would impose an unacceptable safety hazard by participation in the study.
5. Study drug-specific exclusion criteria:
   - For **Aprotinin:**
     - Known active thromboembolic disease, defined as a history of idiopathic (unprovoked) deep vein thrombosis or pulmonary embolism, recent (<3m) deep vein thrombosis or pulmonary embolism, recent (<6m) myocardial infarction or coronary stenting, recent (<6m) ischemic stroke
     - Renal insufficiency with CrCl <30ml/min or continuous renal replacement therapy, hemodialysis, or peritoneal dialysis
     - Recent (<6m) cardiac surgery with cardiopulmonary bypass and/or use of aprotinin

- For high-prophylactic dose of **LMWH**:
  - Active bleeding, a history of intracranial bleeding, or a recent (<3m) GI bleeding requiring transfusion and/or intervention, recent surgery in the central nervous system
  - Renal insufficiency with CrCl < 20ml/min or continuous renal replacement therapy, hemodialysis, or peritoneal dialysis
  - Blood platelet count < 30 000/µL
  - Other conditions that are judged to carry an increased risk of bleeding as judged by the investigator
  - Need for therapeutic anticoagulation (known active thrombo-embolic diseases, atrial fibrillation, mechanical prosthetic heart valve,…)
  - Known hypersensitivity to heparin or LMWH
  - Active bacterial endocarditis
  - Chronic alcoholism
- For **Anakinra:**
  - Impairment of cardiac function defined as severe heart failure, unstable angina pectoris, myocardial infarction within 6 months before enrollment, ventricular arrhythmia requiring treatment or intervention.
  - Severe renal dysfunction (creatinine clearance ≤ 20mL/min) or receive continuous renal replacement therapy, hemodialysis, or peritoneal dialysis.
  - Uncontrolled hypertension (persistent systolic blood pressure >180mmHg, or diastolic blood pressure >110mmHg)
  - Clinical suspicion of latent tuberculosis
  - Clinical suspicion of severe bacterial surinfection (e.g. ventilator-associated pneumonia)
  - Known hypersensitivity to anakinra or to proteins produced by E.Coli

Participants who meet one or more of the above exclusion criteria **must not proceed** to be enrolled/randomized in the Trial and will be identified on the Screening Log as Screen Failure.

Note: Because the intervention of **DAWN-AntiCo** is independent of the background antiviral therapy, any antiviral therapy is allowed, including other investigational agents. Thus, participation in another study, including other DAWN-studies with antiviral agents, does not exclude a patient from randomization within **DAWN-AntiCo**.

3 Trial Procedures

3.1 Participant Consent and withdrawal of consent

The Trial will be conducted only on the basis of prior informed consent by the Trial participants and/or their legally authorized representative(s). As such, no Trial-related procedures will be conducted prior to obtaining written informed consent from potential Trial participants.

When signed informed consent is not permitted because of safety regulations related to the prevention of the transmission of SARS-CoV-2, verbal informed consent shall be documented in the medical records. Signed informed consent shall then be obtained as soon as permitted based on safety regulations to prevent the transmission of SARS-CoV-2.

The process for obtaining and documenting initial and continued informed consent from potential Trial participants will be conducted in accordance with ICH-GCP E6(R2), applicable regulatory requirements and internal Standard Operating Procedures (SOPs).

All originally signed obtained Informed Consent Forms (ICFs) must be retained/archived in the Investigator Site File (ISF) at the Participating Site and must not be destroyed (even when a scanned copy is available) before expiration of the legal archiving term as defined in the protocol section entitled "Archiving".

Participants may voluntarily withdraw consent to participate in the Trial for any reason at any time. The participant's request to withdraw from the Trial must always be respected without prejudice or consequence to further treatment. Consent withdrawal will be documented in the participant's medical

record. The PI must take into account the consequences of such withdrawal: (1) further use of personal data/Trial data, (2) use of human biological materials already collected, (3) safe transition to alternative treatment options, etc. as applicable.

3.2 Selection of Participants / Recruitment

Only adult hospitalized patients diagnosed with COVID-19 will be included.

3.3 Randomization Procedure

A randomization procedure through a computerized system has been established, generated by the data management unit of the clinical trial center Leuven, which ensures the integrity of the trial. Because DAWN-ANTICO does not include an antiviral strategy, participation in this study is possible regardless of background antiviral therapy, including other investigational products.

For the multicentre study a 2 (intervention) to 1 (usual care) will be allocated. Block randomisation (groups of 6 or 9) in every participating center will be implemented.

3.4 Trial Procedures

3.4.1 By visit

Screening:
Patients with documented COVID-19 who require hospitalization will be screened for eligibility. Informed consent will be obtained. When written informed consent is not possible due to restrictions to prevent the transmission of SARS-CoV-2, oral informed consent will be documented in the medical files, and completed with written informed consent as soon as the restrictions do no longer apply. Demographic parameters will be obtained. A medical history will be obtained as part of routine clinical care. When study-related procedures impose an additional burden on the clinical care of patients, they can be waived.

Baseline:
Parameters should be obtained as part of routine clinical care. Baseline lab values will be assessed for the presence of hyperinflammation, as defined in **paragraph 6.1.**
When study-related procedures impose an additional burden on the clinical care of patients, they can be waived. Study drug will be administered when randomized to the investigational drug arm. Medication will be reviewed using the electronic medical files. Serious adverse events and adverse events grade IV will be collected when these are not outcomes of the study.

Daily assessments until discharge:

- Administration of study drug
- Vital signs including SpO2
- Clinical data collection for assessment of study outcomes
- Targeted medication review
- Adverse event evaluation
- Lab values on day 3, 6 and 15, and other only if done as part of routine care;

Serious adverse events and adverse events grade IV will be collected when these are not outcomes of the study. When study-related procedures impose an additional burden on the clinical care of patients, they can be waived.

Visit at Day 15 (+/-2) and 29(+/-5)

These visits can be phone visits when patients are no longer hospitalized.

3.4.2 Laboratory tests

In **DAWN AntiCo**, a blood sample is taken on day 3, 6, and 15. To avoid burden on clinical care in a time of a strained health care system, all other laboratory tests are part of routine clinical care and are not mandatory, but when available will be collected (including but not limited to CRP, white cell count, hemoglobin, platelets, creatinine, hsTroponinT, glucose, total bilirubin, ALT, and AST).

3.4.3 Other investigations

The study includes an optional sample on Day 6 (+- 2): an additional assessment (e.g., nasopharyngeal swab) for SARS-CoV-2 qualitative and quantitative PCR, on the condition that this does not hinder routine clinical care. The sample will be stored and analyzed at a later time point if feasible.

Other additional procedures are:
Plasma sample for assessment of inflammatory cytokines (50 mcl) is included on day 6 and 15, on the condition that this does not interfere with routine clinical care.

3.4.4 Exploratory investigations

A follow-up call on D90 will be organized for survival data.
The study includes the collection of data on a clinical follow-up visit on the condition that this is feasible as part of the routine outpatient follow-up. The patient can visit the ambulatory practice and perform the functional and radiological evaluation that is part of good clinical follow-up. In the case of patients physical condition permits no ambulatory monitoring visit, and an additional call will be organized by the study team for follow-up.

- Clinical examination
- Medication and adverse event review
- Spirometry with reversibility
- Longvolumes and diffusing capacity
- Low dose CT scan
- 6 minutes walk (at the physician's discretion)
- Transthoracic echocardiography (at the physician’s discretion)

3.5 Premature discontinuation of Trial treatment

Participants may voluntarily discontinue Trial treatment and/or prematurely end their participation in the Trial for any reason at any time. In such a case, the Investigator must make a reasonable effort to contact the participant (e.g., via telephone, e-mail, letter) in order to document the primary reason for this decision.

The Investigator may also decide at any time during the Trial, to temporarily interrupt or permanently discontinue the Trial treatment if it is deemed that continuation would be detrimental to, or not in the best interest of the participant.

Similarly, the Sponsor, Ethics Committee or authorized regulatory authority can decide to halt or prematurely terminate the Trial when new information becomes available whereby the rights, safety, and well-being of Trial participants can no longer be assured, when the integrity of the Trial has been compromised, or when the scientific value of the Trial has become obsolete and/or unjustifiable.

Circumstances requiring premature treatment interruption or discontinuation of the Trial, include but are not limited to:

- Safety concerns related to IMP or unacceptable intolerability

- Trial participation while in violation of the inclusion and/or exclusion criteria
- Pregnancy
- Intention of becoming pregnant
- Thrombotic complication (DAWN-AntiCo)
- Bleeding complication (DAWN-AntiCo)
- …

In any such case of early Trial termination and/or treatment interruption/discontinuation, the Investigator will continue to closely monitor the participant's condition and ensure adequate medical care and follow-up. For patients with treatment interruption or discontinuation, all efforts will be made to continue to collect study endpoints, and regular study visits will proceed as per protocol unless patient specifically wishes to discontinue study follow-up.

For participants whose status is unclear because they fail to appear for Trial visits without stating an intention to discontinue or withdraw, the Investigator must make every effort to demonstrate "due diligence" by documenting in the source documents which steps have been taken to contact the participant to clarify their willingness and ability to continue their participation in the Trial (e.g., dates of telephone calls, registered letters, etc.).

A participant should not be considered lost to follow-up until due diligence has been completed.

4 Trial Medication / Drug

DAWN ANTICO

Generic Drug Name<br>(& company brand name)	IMP or non-IMP	Used within Indication? (Y or N)
Aprotinin 500.000 KIUin 50ml iv	IMP	N
LMWH (enoxaparin or fraxiparin) 50IU/kg twice daily in hospitalized patients, 75 IU/kg twice daily in patients on intensive care units (with a minimal dose of 4000IU twice daily)	IMP	Y
Anakinra (solution 100mg/0.67ml)	IMP	N

4.1 Investigational Medicinal Product and Dosing Regimen

The study design is adaptive, to allow the adjustment of a treatment arm/stratum, the addition of a new treatment arm/stratum or the removal of a treatment arm/stratum based on the most updated information in a rapidly evolving field, based on the continuous assessment of the existing evidence available for the IMP and other potential drug candidates.

DAWN AntiCo

This study will randomize patients 2:1 to either a targeted approach to thrombo-inflammatory modulation or standard thromboprophylaxis. This intervention is regardless of the background antiviral strategy, including investigational products.

Standard thromboprophylaxis: In accordance with recent international data about the hypercoagulable state in patients with COVID-19, we use a higher than usual dose of thromboprophylaxis. In hospitalized

patients, 0.5mg/kg enoxaparin (with a minimum of 40mg/d) is given once daily. In patients admitted to an intensive care unit, enoxaparin is given at 0.5mg/kg twice daily (with a minimum of 40mg twice daily). In ICU patients, the dose is reduced by 50% (to 0.5mg/kg once daily) in patients with an estimated CrCl < 30ml/min. Similar doses of other LMWH (fraxiparin) are allowed.

The targeted approach to thrombo-inflammatory modulation;

This interventional arm consists of a standard strategy in all patients, which is based on widely used and broadly available drugs with a well-known safety and efficacy problem. It can be broadly implemented in clinical care. In patients who develop hyperinflammation throughout the study follow-up, a targeted anti-inflammatory treatment will be added. This allows the combination of a cheap and safe intervention in the majority of patients, with the option to add target-specific therapies in a subset of patients.

1. <u>Treatment of hyperfibrinolysis and contact activation:</u>

A. High-prophylactic dose of LMWH

Patients randomized to the intervention arm will receive high-prophylactic doses of LMWH. These doses are below therapeutic doses of LMWH, but higher than usual thromboprophylactic doses.
LMWH will be admininstered at 50IU/kg (e.g; enoxaparin 0.5mg/kg) twice daily, with a minimum of 4000IU (e.g. 40mg enoxaparin) twice daily in non-ICU patients and 75IU/Kg (e.g. enoxaparin 0.75mg/kg) twice daily - with a minimum of 4000IU (e.g. enoxaparin 40mg) - twice daily, in ICU patients.
Twice daily administration will be a morning and evening administration with 12h±2h interval.
The total daily dose will be reduced by 50% in patients with a CrCl<30ml/min (once daily instead of twice-daily administration). In patients who develop severe renal dysfunction (CrCl<15ml/min) or who require renal replacement therapy, administration of LMWH should be interrupted until CrCl returns ≥30ml/min. In patients who develop a CrCl<15ml/min, choice and dose of thromboprophylactic therapy is left to the discretion of the investigator. Options may include unfractionated heparin in a continuous iv infusion with a target aPTT 40-60sec or a target anti-Xa of 0.2-0.4U/ml, and with routine follow-up of aPTT or anti-Xa as per local practice. In patients receiving aprotinin (first 72 hours), aPTT and ACT should not be used to monitor heparin effects, and use of anti-Xa levels is suggested for heparin monitoring.

CrCl		non-ICU	ICU
≥30ml/min		enoxaparin 0.5mg/kg twice daily, with a minimum of 40mg twice daily	enoxaparin 0.75mg/kg twice daily, with a minimum of 40mg twice daily
<30		enoxaparin 0.5mg/kg once daily, with a minimum of 40mg daily	enoxaparin 0.75mg/kg once daily, with a minimum of 40mg daily
<15		enoxaparin 0.5mg/kg once daily, with a minimum of 40mg daily	Stop study LMWH. Consider unfractionated heparin iv infusion with target APTT 40-60 sec or target anti-Xa of 0.2-0.4U/ml (at the investigator's discretion, not part of the study medication). In patients receiving aprotinin, aPTT

			should not be used to guide heparin dosing

Equivalent doses of other LMWH (fraxiparin) can be used as per local availability

B. Aprotinin

Additionally, to suppress hyperfibrinolysis and the bradykinin-kallikrein pathway, patients will receive aprotinin. Aprotinin will be administered as an iv infusion of 2 MIE over 30 minutes four times per day from day 0 to day 3. Prior to the first administration, a test dose of 1ml of a 50ml vial will be given, with control of vital parameters during 20 minutes, before proceeding to full study dose.
Instruction for test dose: after iv injection of 1 ml of aprotinin, patient vital parameters will be monitored continuously (if on ICU) or with 5-minute intervals if not on ICU. During the test dose, patient will be supervised clinically and monitored for signs or symptoms of allergic reaction. Rescue medication to treat anaphylaxis, including H1- and H2-antihistaminergic drugs and adrenalin, should be immediately available.
Adminitration of aprotinin will be at 6-hour intervals, with the first administration together with first administration of study LMWH.
Because aprotinin inhibits kallikrein, which is central in the activation of the intrinsic pathway of the coagulation cascade, the aPTT will be prolonged following its administration, reflecting its pharmacodynamics effects. Because patients also receive anticoagulation to protect these patients against the frequently observed thrombotic events (see also session on Anticoagulation / Low Molecular Weight Heparins), it is important that the aPTT not be used to assess the anticoagulant effects of heparins. If ACT is used to monitor coagulation in patients treated with trasylol, it should be noted that CELITE ACT test can be falsely elevated in patients treated with aprotinin, whereas aprotinin does not interfere with kaolin-based ACT tests.
Aprotinin should not be coadministered with other drugs; a separate iv line is required.

2. <u>Treatment of hyperinflammation:</u>

Patients in the intervention arm will be monitored for hyperinflammation. If at any point during the study, the patient develops signs of hyperinflammation, treatment with anakinra will be started, unless the patient has a contraindication for anakinra (any of the following):

- Impairment of cardiac function defined as severe heart failure, unstable angina pectoris, myocardial infarction within 6 months before enrollment, ventricular arrhythmia requiring treatment or intervention.
- Severe renal dysfunction (creatinine clearance ≤ 20mL/min) or receive continuous renal replacement therapy, hemodialysis, or peritoneal dialysis.
- Uncontrolled hypertension (persistent systolic blood pressure >180mmHg, or diastolic blood pressure >110mmHg)
- Clinical suspicion of latent tuberculosis

In patients receiving anakinra who develop ventricular arrhythmia requiring intervention, uncontrolled hypertension, or severe renal dysfunction or renal replacement therapy, anakinra infusion will be stopped.

Presence of of hyperinflammation is defined as:

a. Absolute lymphocyte count < 1000 cells/mL
b. Two of the following: i. Ferritin > 800ng/mL ii. LDH > 400 U/L iii. D-Dimers > 1000 ng/mL

These lab values have been identified as independent predictors of adverse outcome in patients with severe COVID-19[1,6,12,35].

Anakinra is delivered as a sterile solution for injection, prefilled in a single-use syringe with a strength of 100 mg. The total volume of injection is 0.67 mL, and the concentration of anakinra in the solution is 150 mg/mL. Anakinra must be stored at refrigerated conditions at 2-8 °C (36°-46°F) in a secure area at the

study sites. Further instructions for handling and storage of the IMP anakinra are available in the IMP manual (cf appendix. Swedish Orphan Biovitrum AB (publ) SE-112 76 Stockholm Sweden is the marketing authorizations holder.

Anakinra is approved for the chronic treatment of several inflammatory diseases as a subcutaneous treatment (at doses of 100 mg/day or in weight-based doses of up to 8 mg/kg/day). The IV administration of anakinra has been studied in clinical trials in healthy volunteers, and critically ill patients with sepsis and hyper-inflammation at variable IV doses up to 3500 mg/day. No safety concerns emerged in these studies[36-39]. A study in children with systemic-onset juvenile arthritis complicated by refractory macrophage activation syndrome is currently ongoing (NCT02780583) in which anakinra is administered at a dose of 10 mg/kg/day to a maximum of a dose of 200 mg/day divided every 12 hours (for children ≤40 kg) or 5 mg/kg/day up to a maximum dose of 400 mg/day divided every 6 hours (children > 40 kg and adults). This dosing schema reflects the dose at which anakinra is administered, although off label, in children affected by this severe disease, as reported in several case studies.

Anakinra will be administered by IV infusion at a total dose of 400 mg per day, divided into 4 doses 100 mg IV every 6 hours. Anakinra treatment will continue for 15 days, i.e., Days 1 to 15. Before administration, the full content of the prefilled, single-use syringe (anakinra 100 mg) will be diluted in 100 mL saline. The IV administration of anakinra has to occur immediately after the preparation over an infusion period of 60 minutes. Full instructions for the preparation of anakinra are available in the IMP manual.

4.2 Concomitant / Prohibited Medication / Treatment

There are currently no approved treatments for COVID-19. Patients will receive the standard of care as continuously updated by national and international guidance.

4.3 Use of rescue anti-inflammatory therapy

In patients with persisting poor clinical status, including but not limited to continued need for mechanical ventilation or further clinical deterioration during the trial, the use of anti-inflammatory drugs (such as corticosteroids, IL-1 or IL-6 blockers,… - non limitative) is permitted at the discretion of the treating physician. Use and modalities of rescue drugs will be recorded.

5 Safety

5.1 Specification, timing, and recording of safety parameters

- Grade 4 adverse events (life-threatening or urgent intervention required)
- SAEs.

- Lab values: CRP, white cell count, hemoglobin, hsTroponinT, platelets, creatinine, glucose, total bilirubin, ALT, and AST on days 1; 3, 5, 8, 11, 15 and 29 (If measured according to clinical indication).

DAWN AntiCo
Modulation of hyperfibrinolysis can increase the risk of thrombosis. Therefore, patients also receive high-dose thromboprophylaxis. If clinical signs of thrombosis develop, IMP can be interrupted/discontinued at the discretion of the investigator. In the case of thrombosis or if another indication for therapeutic anticoagulation emerges during follow-up (e.g., atrial fibrillation), therapeutic anticoagulation should be initiated as per local standard care.

Anticoagulation can increase the risk of bleeding. For patients who develop active bleeding or who are at high risk of bleeding as judged by the investigator, e.g., because of drop-in platelet counts, IMP can be

interrupted/discontinued at the discretion of the investigator and treatment of the bleeding should be done as per local standard care.

Aprotinin has a risk of IgG-mediated anaphylactic reactions. If at any time, a suspected anaphylactic reaction occurs, use of aprotinin will be interrupted.

5.1.1 Definitions

Adverse Event (AE)

An AE is any untoward medical occurrence in a patient or subject during an experiment and does not necessarily have a causal relationship with this treatment.

An AE can, therefore, be any unfavorable and unintended sign (including an abnormal laboratory finding), symptom, or disease temporally associated with the use of a product, whether or not considered related to the product. Any worsening (i.e., any clinically significant adverse change in the frequency or intensity of a pre-existing condition) should be considered an AE.

Adverse Reaction (AR) or Adverse Drug Reaction (ADR)

An AR is any untoward and unintended responses to an investigational medicinal product or to an experiment and when an investigational product is concerned, related to any dose administered.

Serious Adverse Event (SAE)

An SAE is any untoward medical occurrence that results in any of the following:

- Death
- A life-threatening[a] experience
- In-patient hospitalization or prolongation of existing hospitalization
- A persistent or significant disability or incapacity
- A congenital anomaly or congenital disability
- Important medical events that may be considered an SAE when - based on appropriate medical judgment - they may jeopardize the subject and may require medical or surgical intervention to prevent one of the above outcomes

[a] The term "life-threatening" in the definition of SAE refers to an event in which the subject was at risk of death at the time of the event. It does not refer to an event which hypothetically might have caused death if it was more severe.

Suspected Unexpected Serious Adverse Reaction (SUSAR)

A SUSAR is an adverse reaction, the nature or severity of which is not consistent with the information on the experiment, and, when a clinical trial is concerned, with the applicable product information (e.g., investigator's brochure for an unauthorized investigational product or the patient leaflet joined to the summary of product characteristics for an authorized product).

5.1.2 Adverse Events that do not require reporting

In general, the following should <u>not be reported as AEs:</u>

- Pre-existing conditions, including those found as a result of screening (these should be reported as medical history or concomitant illness).
- Pre-planned procedures, unless the condition for which the procedure was planned, has worsened from the first trial-related activity after the subject has signed the informed consent.

The following events <u>not to be considered as SAEs</u> are:

- Pre-planned hospitalizations unless the condition for which the hospitalization was planned has worsened from the first trial-related activity after the subject has signed the informed consent.
- Hospitalization as part of a standard procedure for protocol therapy administration. However, hospitalization or prolonged hospitalization for a complication of therapy administration will be reported as an SAE.
- Hospitalization or prolongation of hospitalization for technical, practical, or social reasons, in the absence of an AE.

For this trial, only Adverse Events graded as severe shall be collected, i.e., adverse events that are life-threatening and/or require urgent intervention. Adverse events that are also outcomes of the trial are also exempt from reporting.

5.1.3 Recording and reporting of Adverse Events

Investigators will seek information on AEs during each patient contact. All events, whether reported by the patient or noted by trial staff, will be recorded in the patient's medical record and the (e)CRF within a reasonable time after becoming aware. If available, the diagnosis should be reported on the AE form, rather than the individual signs or symptoms. If no diagnosis is available, the Investigator should record each sign and symptom as individual AEs.

The following minimum information should be recorded for each AE:

- AE description
- start and stop date of the AE
- severity
- seriousness
- causality assessment to the Investigational Medicinal Product (IMP) and/or study procedures
- outcome

5.1.4 Assessment

All AEs must be evaluated by an Investigator as to:

- **Seriousness:** whether the AE is an SAE. See above for the seriousness criteria.
- **Severity:**
    - Severity must be evaluated by an Investigator according to the following definitions:
        - *Mild* – no or transient symptoms, no interference with the subject's daily activities
        - *Moderate* – marked symptoms, moderate interference with the subject's daily activities
        - *Severe* – considerable interference with the subject's daily activities, unacceptable
- **Causality:**
    - *None* – An AE which is not related to the IMP or experiment
    - *Unlikely* – An AE for which an alternative explanation is more likely (e.g., concomitant medication(s), concomitant disease(s)), and/or the relationship in time suggests that a causal relationship is unlikely
    - *Possible* – An AE which might be due to the use of the IMP or the experiment. An alternative explanation is inconclusive. The relationship in time is reasonable; therefore, the causal relationship cannot be ruled out.
    - *Probable* - An AE which might be due to the use of the IMP or the experiment. The relationship in time is suggestive (e.g., confirmed by dechallenge). An alternative explanation is less likely.
    - *Definitely* – An AE which is listed as a possible adverse reaction and cannot be reasonably explained by an alternative explanation. The relationship in time is very suggestive (e.g., it is confirmed by dechallenge and rechallenge).

5.1.5 Timelines for reporting

For this trial, only Adverse Events grade 4 shall be collected, i.e., an adverse event that is life-threatening and/or requires an urgent intervention. Adverse events that are also collected as outcomes will not be separately reported.

All SAEs must be reported to the Sponsor within 24 hours of the trial staff becoming aware of the event. The immediate report shall be followed by detailed, written reports. The immediate and follow-up reports shall identify subjects by Trial identification.

SAE details will be reported by the Investigator to the Sponsor:

- By completing the SAE form in the (e)CRF

5.1.6 Follow-up

The Investigator must record follow-up information by updating the patient's medical records and the appropriate forms in the (e)CRF. The worst-case severity and seriousness of an event must be kept throughout the trial.

SAE follow-up information should only include new (e.g., corrections or additional) information and must be reported within 24 hours of the Investigator's first knowledge of the information. This is also the case for previously non-serious AEs, which subsequently become SAEs.

- All *SAEs* must be followed up until the outcome of the event is 'recovered', 'recovered with sequelae', 'not recovered' (in case of death due to another cause) or 'death' (due to the SAE) and until all related queries have been resolved, or until end of the trial (whichever occurs first).
- *Non-serious AEs* must be followed up until the patient's last study visit, and until all related queries have been resolved.

SAEs after the end of the trial: If the Investigator becomes aware of an SAE with a suspected causal relationship to the IMP or experiment after the subject has ended the trial, the Investigator should report this SAE within the same timelines as for SAEs during the trial.
For women with child-bearing potential, the use of highly effective contraception is required for three months after last study dose.

5.1.7 Death

All deaths will be reported without delay to the sponsor (irrespective of whether the death is related to disease progression, the IMP, study procedure, or is an unrelated event). The sponsor will notify all deaths as soon as possible after becoming aware of the Central EC and the EC of the concerned site and provide additional information if requested.

5.1.8 Reporting requirements to Ethics Committee's (EC's) and Competent Authorities (CA's)

The Investigator is responsible for ensuring that all safety events are recorded in the (e)CRF and reported to the Sponsor following instructions provided below.

The Sponsor will promptly evaluate all SAEs against medical experience to identify and expeditiously communicate possible new safety findings to Investigators, EC's, and applicable CA's based on applicable legislation.

5.1.9 Sponsor's reporting of Suspected Unexpected Serious Adverse Reactions (=SUSARs)

After receiving the SAE report form from the Investigator, the Sponsor has to make a causality (relationship) assessment. The term SADR (Serious Adverse Drug Reaction) is to be used whenever either the Investigator or the Sponsor deems the SAE as possibly or probably related to the IMP.

The Sponsor must evaluate (and document the evaluation of) the expectedness for each SADR against the Reference Safety Information, e.g., in the Investigator's Brochure or applicable product information. In case the event is Unexpected (= a SUSAR) it must be reported by the Sponsor to the EC's, CA's (through the EudraVigilance database) and other participating Investigators within the following timelines:

- **7** calendar days if a fatal or life-threatening event (follow-up information within an additional 8 days)
- **15** calendar days if a non-fatal or non-life-threatening event (follow-up information as soon as possible)

For reporting to the EudraVigilance database, all information related to the SUSAR should be provided by the Sponsor to the CTC of UZ Leuven as soon as possible. Contact details: CTC@uzleuven.be and tel. 016 34 19 98.

5.1.10 Annual reporting

The Sponsor has an obligation to, once a year throughout the clinical trial (or on request), submit a progress report to the EC's and CA's containing an overview of all SARs occurred during the reporting period and taking into account all newly available safety information received during the reporting period.

5.1.11 Data and Safety Monitoring Board (DSMB) and Treatment stopping rules

Due to the exceptional circumstances, namely a pandemic of the SARS-CoV-2 virus, and the urgency with which this DSMB has been assembled, DSMB members are not independent from the Sponsor. For further information see separate document DSMB Charter for "DAWN-azithromycine" COVID-19 trial (S63935) (v1 15 April 2020)

Given the severity of illness in COVID-19, there are no pre-specified study stopping rules for safety. The protocol team will review AE / SAE data on an ongoing basis. If there are a concerning number of unexpected AEs, the DSMB will be asked to review safety data in an ad hoc meeting.

The DSMB will review safety data after 80 subjects are entered into the trial and ad hoc reviews will be undertaken if there are other specific safety concerns. The study will not stop enrolment awaiting these DSMB reviews, though the DSMB may recommend temporary or permanent cessation of enrolment based on their safety reviews. There are no pre-specified treatment stopping rules.

Their interim monitoring will allow early stopping for safety, or if new effective therapies are identified through these trials and should become standard of care immediately, in an attempt to control the COVID-19 pandemic as quickly as possible.

5.1.12 Communication plan to report relevant safety findings to all stakeholders

Findings of the DSMB: immediate communication by the Coordinating Investigator or his delegate to all study investigators and relevant study personnel

Ongoing review of safety information will be performed by the Coordinating Investigator, in case of unexpected trends this information will be reviewed by the DSMB
All unexpected events which affect the benefit-risk balance of the clinical trial, but are not suspected unexpected serious adverse reactions, will be reported by the Coordinating Investigator to the FAHMP, ethics committees and study investigators as soon as possible, but no later than 15 days from the date the sponsor became aware of this event.

Urgent safety measures: Where an unexpected event is likely to seriously affect the benefit-risk balance, the sponsor shall take appropriate urgent safety measures to protect the subjects. These will be reported by the Coordinating Investigator or his delegate as soon as possible to the FAHMP and ethics committees but no later than seven calendar days from the date the measures have been taken.

Relevant new safety information will be communicated to the trial subjects by means of an update to the informed consent form or any other communication pre-approved by the ethics committee.

SUSAR: if an SAE is reported in the eCRF, an automatic email notification is sent to the Coordinating Investigator and Safety reviewer of the CTC of UZ Leuven. In case the reporting Investigator assesses the event to be possibly, probably or definitely causally related to the study medication, the Coordinating Investigator will evaluate the expectedness of the event based on the Reference Safety Information. This information will be recorded in the eCRF as soon as possible, preferably within the same working day. In case of a SUSAR, the reporting Investigator will be contacted by the Coordinating Investigator and asked to provide all relevant information related to the event to the Coordinating Investigator and CTC, using

the CIOMS template, within 3 working days. The CTC will report the event to the FAHMP (via EudraVigilance) and ethics committees within 3 working days. The Coordinating Investigator will report the event to all study investigators and relevant study personnel.

6 Statistics and Data Analysis

Statistical analysis will be performed in accordance with ICH E9; a detailed description of the analysis is provided in the separate Trial-specific Statistical Analysis Plan (SAP). ICH E3 and E8 will guide the structure and content of the clinical trial report. A brief summary is provided here. Details will be described in the SAP. The general statistical approach of the DAWN study can still be revisioned, as it is subject to the development of future treatment strata.

General considerations:

Adaptive design and blinded interim analysis

This study is intended to allow for two types of adaptations:

1) blinded confirmation or modification of the day selected for the primary outcome analysis and

2) the ability to add a new experimental arm or stratum if one becomes available.

Blinded endpoint confirmation or modification

The current plan is to evaluate the primary outcome on Day 15, in line with the WHO master protocol. Because there is uncertainty about the clinical course and different potential trajectories according to baseline disease severity, the day of the primary outcome may be modified based on a blinded evaluation of various time points (e.g., days 7-21)[19]. This will occur after approximately 100 participants have been enrolled by a blinded endpoint evaluation committee without knowledge of treatment assignment. Analyses will be evaluated by baseline severity (mild/moderate vs. severe). For example, in mild disease, recovery may occur rapidly such that all with mild disease have resumed normal activities by Day 15. Hence, the final timepoint selected may vary accordingly.

Addition of new experimental therapies

If additional data become available to add an experimental therapy, analyses of experimental arms/strata will be performed comparing concurrently enrolled control subjects. If one treatment crosses an efficacy stopping boundary, this treatment may become the new control arm for comparisons. This approach was used in the recent PALM Ebola therapy RCT[20].

Primary outcome

For the pilot phase of **DAWN AntiCo,** to evaluate the effect of modulation of thrombo-inflammation, we will evaluate D-dimers on day 6 as a separate primary outcome. The null hypothesis being tested is that the mean D-dimer level on day 6 is the same for the standard of care and intervention arm.

For the **overall DAWN** study program, the primary outcome is based on an ordinal severity scale with 7 categories. This scale has been proposed by the WHO for COVID-19 related research and has been previously used in trials of patients with influenza. Previously reported studies and ongoing studies record the same primary outcome, which allows cross-study data pooling.

Primary outcome is the time from Day 0 to sustained clinical improvement or life discharge, whichever comes first, whereby a sustained clinical improvement is defined as an improvement of $\geq$ 2 points vs the highest value of Day 0 and 1 and sustained for at least 3 days.

The null hypothesis being tested is that the primary outcome is the same for the standard of care and experimental treatment arms.

6.1 Sample Size Determination

Despite the rapid dissemination of data from clinical case series and some early-stage clinical trials, detailed information about the course of the disease is limited in this stage of the COVID-19 pandemic. The sample sizes presented here are only illustrative. The larger the number randomized the more accurate the results will be, but the numbers that can be randomized will critically depend on how large the epidemic becomes.

Furthermore, in the absence of treatments with a known benefit, rapid changes in standard of care are to be expected, and important signs of a benefit or harm of treatment under investigations will require rapid reporting. The interim trial results will be monitored by a Data Monitoring Committee. If at any stage evidence emerges that any one treatment arm/stratum is inferior, then it can be decided that that arm/stratum will be discontinued. Conversely, if good evidence emerges while the trial is continuing that some other treatment(s) should also be being evaluated, then it can be decided that one or more extra arms or strata will be added while the trial is in progress.

Sample size estimates provided as a reference but not to indicate the final number of patients to be randomized.

Pilot phase DAWN AntiCo

In the pilot phase of DAWN AntiCo, we will evaluate the effect of modulation of the thromboinflammatory response on biochemical parameters. To evaluate the effect of this strategy, we will evaluate the difference in mean D-dimers on day 6.

Published data show highly variable D-dimer values in patients with COVID. For an estimated mean D-dimer of 1000 with a standard deviation of 600, a sample size of 46 patients (1:1 randomization) has a power of 0.8 and an alpha of 0.05 to detect a difference of 500. We will evaluate the effect of the intervention after a pilot phase of 2x25 patients. Based on the information from other DAWN studies, the final sample size for effect on clinical outcomes will be evaluated following this pilot phase.

In their study comparing clinical improvement rates for Lopinavir-Ritonavir in hospitalized patients with severe Covid-19, Cao et al reported a clinical improvement rate in the control group of 37.7% on Day 14 [21]. Therefore, for our sample size calculations, we assume that a 40% improvement rate will be observed at Day 15 in the control group. Based on the log-rank test, with a 2-sided significance level of 5% and 80% statistical power and using a (2:1) randomization ratio in favour of the intervention, we estimate that a total sample size of 354 patients will suffice to detect an absolute improvement of 15% (i.e. 55% in intervention group). To detect an absolute improvement of 20% (60% in intervention group), a total sample of 196 patients will suffice. We propose a pragmatic sample size of 210 patients taking into account early dropouts.

6.2 Statistical Analysis

6.2.1 Population for analysis

The following analysis sets will be defined:

Full Analysis Set (FAS): The FAS will include all randomized patients according to their randomized treatment. Patients randomized to the interventional group will be excluded if they did not receive any dose of study medication. The FAS will be used for the evaluation of all efficacy endpoints. As described in inclusion criterium 4b, patients without PCR-confirmed diagnosis at inclusion in whom despite all efforts, the diagnosis of COVID-19 could not be confirmed (by serology, PCR on BAL, or other approved diagnostic test) will be excluded from the analysis.

Safety Set (SS): The SS will include all patients who were randomized according to their actual treatment. Patients randomized to the interventional group who did not receive any study treatment will be included in the Standard Of Care group, The SS will be used for the evaluation of all safety parameters.

6.2.2 Statistical Analyses

6.2.2.1 General Approach

This is an open-label controlled randomized trial testing a superiority hypothesis with a two-sided type I error rate of 0.05. In this exploratory study, secondary hypotheses will be tested in a non-hierarchical way. These will be described according to the appropriate summary statistics (e.g., proportions for categorical data, means with 95% confidence intervals for continuous data, median for time-to-event data).

A statistical analysis plan (SAP) will be developed and filed with the study sponsor before database lock.

6.2.2.2 Analysis of the Primary Efficacy Endpoint

The primary will be analysed by means of competing risk analyses whereby death without any improvement will be considered as a competing risk.

Event rates will be estimated using cumulative incidence functions (CIF). Median times to improvement will be calculated by treatment group.

The effect of treatment will be assessed by performing a Fine&Gray competing risk regression model that includes the baseline value on Day 0 as a covariate and randomised treatment as a factor. From the Fine&Gray model, the treatment effect and associated 95% confidence interval will be estimated

In the pilot phase of **DAWN-ANTICO**, the effect of modulation of thrombo-inflammation will be evaluated on a laboratory primary efficacy endpoint, mean D-dimer level at day 6. The null hypothesis being tested is that the mean D-dimer level on day 6 is the same for the standard of care and intervention arm. Means will be compared with appropriate statistical testing after checking for normality. The details of the analysis are given in a separate statistical analysis plan.

To evaluate the effect of TXA and high-dose enoxaparin vs. anakinra, analyses will be performed separately in patients with or without signs of hyperinflammation (i.e., with or without an indication for anakinra in the intervention group).

6.2.2.3 Analysis of the Secondary Endpoint(s)

1. Cumulative clinical status up to Day 15 will be analysed using a general linear model adjusted for clinical status on Day 0. The treatment effect will be estimated by the difference of mean values between the groups.

2. Cumulative clinical status recorded daily during hospital stay and on Days 15 and 19 will be analysed by means of a proportional odds logistic regression model, adjusted for clinical status on Day 0. The treatment effect will be estimated by the common odds ratio.
3. All-Cause mortality rates will be estimated by the treatment group using the Kaplan-Meier method. The resulting Kaplan-Meier curves will be compared using a log-rank test. The treatment effect will be estimated by the hazard ratio using a Cox regression.
4. Time-to-event parameters with competing risk (time to clinical improvement, composite cardiac endpoint): event rates will be estimated using cumulative incidence functions (CIF), the resulting CIF curves will be compared using Gray's test. The treatment effect will be estimated by the subdistribution hazard ratio.
5. Duration of hospital and ICU stay: both parameters will be analyzed as time-to-event parameters with competing risk, whereby the event of interest is discharged from hospital/ICU, and the competing risk is hospital/ICU death.
6. Continuous normally distributed variables (e.g., QTc) will be analyzed using a 2-sample t-test. Treatment effects will be estimated by the difference in mean values between the groups. If applicable, changes from baseline will be calculated. Comparisons between treatment groups will be made by performing an analysis of covariance (ANCOVA) on the post-baseline value, using the baseline value as a covariate.
7. Continuous non-normally distributed variables (clinical status, NEWS score, duration of supplemental oxygen, duration of mechanical ventilation) will be analyzed using a Wilcoxon rank-sum test. Change in ordinal scale at specific time points will be compared using Wilcoxon rank-sum tests.
8. The proportion of patients with D-dimer <1000, ferritin <1000, and LDH <600 on day 6 and day 15 will be compared.
9. The proportion of patients who develop hyperinflammation at day 3, 6, and 15; time to hyperinflammation

Missing data procedures will be described in the SAP.

6.2.2.4 Safety Analyses

Safety endpoints are described above. These events will be analyzed univariately and as a composite endpoint. Time-to-event methods will be used for death and the composite endpoint. Each AE will be counted once for a given participant and graded by severity and relationship to COVID-19 or study intervention.

Adverse events leading to premature discontinuation from the study intervention and serious treatment-emergent AEs will be described as part of the primary publication of the study results.

6.2.2.5 Baseline Descriptive Statistics

Baseline characteristics will be summarized by treatment arm/stratum. For continuous measures, the mean and standard deviation will be summarized. Categorical variables will be described by the proportion in each category (with the corresponding sample size numbers).

6.2.2.6 Planned Interim and Early Analyses

Early analysis

An initial blinded endpoint-evaluation phase will be enrolled prior to specification of the primary endpoint as described above. Analysis and decision making will be restricted to a blinded endpoint evaluation committee (DSMB). DSMB membership will be defined elsewhere and will consist only of individuals who

are blinded to treatment assignment. Principles of blinded endpoint-evaluation will be defined in a separate document.

Additional early analyses include monitoring enrolment, baseline characteristics, and follow-up rates throughout the course of the study by the study team. Analyses will be conducted blinded to treatment assignment.

In **DAWN AntiCo**, analysis of the primary laboratory endpoint in the pilot phase is planned after 50 patients.

Interim analyses

An data and monitoring safety board (DSMB) will monitor ongoing results to ensure patient well-being and safety as well as study integrity. The DSMB will be asked to recommend early termination or modification only when there is clear and substantial evidence of a safety issue.

6.2.2.7 Sub-Group Analyses

Subgroup analyses for the primary and selected secondary outcomes will evaluate the treatment effect across the following subgroups: duration of symptoms prior to enrolment, age groups, disease severity at baseline and co-morbidities. A forest plot will display confidence intervals across subgroups. Interaction tests will be conducted to determine whether the effect of treatment varies by subgroup.

Data and monitoring safety board (DSMB)
The DSMB will review safety data after 80 subjects are entered into the trial and ad hoc reviews will be undertaken if there are other specific safety concerns. The study will not stop enrolment awaiting these DSMB reviews, though the DSMB may recommend temporary or permanent cessation of enrolment based on their safety reviews.
Given the severity of illness in COVID-19, there are no pre-specified study stopping rules for safety. The protocol team will review AE / SAE data on an ongoing basis. If there are a concerning number of unexpected AEs, the DSMB will be asked to review safety data in an ad hoc meeting.

Their interim monitoring will allow to recommend early stopping for reasons of safety. If new effective therapies are identified through these trials, these snould become standard of care immediately, in an attempt to control the COVID-19 pandemic as quickly as possible.

Because of the exceptional circumstances, the DSMB is part of UZ Leuven and cannot be considered as fully independent. A charter and terms of reference have been provided to make sure scientific independency of the DSMB members has been sufficiently assured.

7 Data handling

7.1 Data Collection Tools and Source Document Identification

Data collection, handling, processing, and transfer for this Trial will be performed in compliance with applicable regulations, guidelines for clinical trials and internal procedures, as follows:

7.1.1.1 Data collection

Source Data will be collected and recorded in the Trial participant's files/medical records.

Worksheets may be used for capturing some specific data in order to facilitate completion of the eCRF. Any such worksheets will become part of the Trial participant's source documentation and will be filed together with or as part of the medical records (during but also following completion of the Trial).

It remains the responsibility of the Investigator to check that all data relating to the Trial, as specified in the Trial protocol, are entered into the eCRF in accordance with the instructions provided and that the forms are filled out accurately, completely and in a timely manner.

eCRFs are provided by the Sponsor for each participant. The Trial data will be transcribed from the source records (i.e. participant's medical file or Trial-specific source data worksheets) into an eCRF by Trial Staff. Transcription to the eCRF will be done as soon as possible after a participant visit and in a pseudonymized manner using a unique identifier assigned by the Sponsor.

The eCRFs will be available for review at the next scheduled monitoring visit (as applicable).

7.1.1.2 Data Validation

All data relating to the Trial must be prepared and validated by the Investigator. Any eCRF entries, corrections and alterations must be made by the Investigator or other authorized Trial staff.

Proper audit trails are available in REDCap to demonstrate the validity of the Trial data collected. This includes historical records of original data entries, by whom and when the data was entered, as well as detailed records of any corrections or additions made to the original data entry (i.e. who made the correction/addition, when and why), without obliterating the original data entry information.

7.1.1.3 Data Management

The Trial Data Manager will perform extensive consistency checks on the received data. Queries will be issued in case of inconsistencies in accordance with internal procedures. A Data Management Plan will be developed to map data flows, data validation measures that will be taken, how (interim) database lock(s) will be managed and, as applicable, the role and responsibilities of the Data Monitoring Committee (DMC)

7.1.1.4 Data Transfer

Any participant records or datasets that are transferred to the Sponsor or any partners of the Sponsor will contain the Trial-specific participant identifier only; participant names or any information which would make the participant identifiable will not be transferred. All pseudonymized data relating to the Trial must be transmitted securely to the Sponsor (see 8.1.2. legal requirements).

7.1.2 Legal requirements

All source data will be kept at a secured location with restricted access at all times. These data must be collected and processed with adequate precautions to ensure confidentiality and compliance with applicable data protection laws and regulations and, more in particular, the EU General Data Protection Regulation 2016/679 (GDPR) and relevant national laws implementing the GDPR. Appropriate technical and organizational measures to protect the data against unauthorized disclosure or access, accidental or unlawful destruction, or accidental loss or alteration must be established. Trial staff whose responsibilities require access to personal data agree to keep the data confidential.

The Investigator and the Participating Site(s) (as applicable) shall treat all information and data relating to the Trial disclosed to them as confidential and shall not disclose such information to any third parties or use such information for any purpose other than the objectives of the Trial as described in this protocol.

The collection, processing, and disclosure of personal data, such as participant health and medical information, are subject to compliance with applicable laws and regulations regarding personal data protection and the processing of personal data.

The Investigator will maintain all source documents and completed eCRF that support the data collected from each Trial participant, and will maintain a Trial Master File (TMF) containing all Trial documents as specified in ICH-GCP E6(R2) Chapter 8 entitled "Essential Documents for the Conduct of a Clinical Trial", and as specified by applicable regulatory requirement(s).
The Investigator will take appropriate measures to prevent accidental or premature destruction of these documents.

Transfer of the pseudonymized data will be performed via a secured method of the transfer, taking into account all appropriate security arrangements and regulations (such as the European General Data Protection Regulation). The receiving party will be bound by contractual agreement to keep the transferred data confidential at all times and to only process the data for the Trial. To this end, appropriate Data Transfer Agreements (DTAs) will be established.

7.2 Audits and Inspections

The Investigator will permit direct access to Trial data and documents for monitoring, audits and/or inspections by authorized entities such as but not limited to: the Sponsor or its designees and competent regulatory or health authorities. As such, eCRFs, source records, and other Trial related documentation (e.g., the Trial Master File, pharmacy records, etc.) must be kept current, complete, and accurate at all times.

7.3 Monitoring

Following ICH-GCP E6(R2) the Sponsor is responsible for monitoring the Trial to ensure compliance with GCP and current legislation, and to verify, among other requirements, that proper written informed consent has been obtained and documented, that the Trial procedures have been followed as shown in the approved protocol. That relevant Trial data have been collected and reported in a manner that assures data integrity. To this end, Source Data will be compared with the data recorded in the eCRF. Monitoring of the Trial will be performed by qualified individuals (independent from the site Trial staff) according to the monitoring plan. The Sponsor and Investigator/Participating Site will permit direct access to the Trial data and corresponding Source Data and to any other Trial related documents or materials to verify the accuracy and completeness of the data collected. More details about the monitoring strategy are described in the Trial specific Monitoring Plan (MP).

7.4 Archiving

As specified in ICH-GCP E6(R2) Section 8.1 Addendum, the Sponsor and Investigator/Participating Site will maintain a record of the location(s) of all respective Essential Trial Documents (including but not limited to Source Documents, completed and final eCRF and ISF(s)/TMF). The Sponsor should ensure that the Investigator has control of and continuous access to the eCRF data reported to the Sponsor during the Trial.

The Investigator/Participating Site should have control of all Essential Documents and records generated by the Investigator/Participating Site before, during, and following termination of the Trial.

The Sponsor is responsible for archiving Trial specific documentation (such as but not limited to the Trial protocol, any amendments to it, the final Clinical Study Report (CSR), and the Trial database) according to ICH-GCP E6(R2). Source data and site-specific Trial documents (such as but not limited to the original signed ICFs) will be archived by the participating site(s) according to local practice, and for at least 25 years following termination of the Trial. Archived data may be held on the electronic record, provided that media back-up exists, hard copies can be obtained, if required, and measures are taken to prevent accidental or premature loss or destruction of data. Destruction of Essential Documents will require written authorization from the Sponsor.

8 Ethical and Regulatory Considerations

8.1 Ethics Committee (EC) review & reports

Before the start of the Trial, this protocol and other related documents (e.g., ICF, advertisements, IB, etc.) will be submitted for review to the EC and the relevant CA for Trial authorization. The Trial shall not commence until such approvals have been obtained.

It is the responsibility of the CI to produce the Annual Progress Report (APR) and submit to the EC/CA within 30 days of the anniversary date on which favorable opinion to start the Trial was given, and annually until the Trial is declared ended.

The CI shall notify the EC/CA of the end of the Trial. Should the Trial be ended prematurely, the CI will notify the EC/CA and include the reasons for premature termination within 15 days of the decision. The CI will submit a final report with the results, including any publications/abstracts, to the EC/CA within 1 year or 6 months for pediatric Trials.

8.2 Regulatory Compliance

The Trial will be conducted in compliance with the principles outlined in the requirements for the conduct of clinical Trials in the EU as provided for in Directive 2001/20/EC or EU Regulation 536/2014, as applicable, and any subsequent amendments, as well as in compliance with ICH-GCP E6(R2) guidelines, other GxP guidelines, the most recent version of the Declaration of Helsinki, the Belgian law of May 7th 2004 regarding experiments on the human person (as amended) or the Belgian law of May 7th 2017 on clinical Trials with medicinal products for human use, as applicable, and with the EU General Data Protection Regulation 2016/679 (GDPR), the relevant Belgian laws implementing the GDPR, the Belgian Law of August 22nd 2002 on patient rights and all other applicable legal and regulatory requirements.

8.3 Protocol / GCP compliance

The Trial must be performed following the protocol, current ICH-GCP guidelines, and applicable regulatory and country-specific requirements. GCP is an international ethical and scientific quality standard for designing, conducting, recording, and reporting studies that involve the participation of human participants. Compliance with this standard provides public assurance that the rights, safety, and well-being of Trial participants are protected, consistent with the principles that originated in the most recent version of the Declaration of Helsinki, and that the Trial data are credible, reliable and reproducible.

The Investigator and Trial team acknowledge and agree that prospective, planned deviations or waivers to the protocol are not permitted under applicable regulations on clinical studies. However, should there be an accidental protocol deviation, such deviation shall be adequately documented in the source documents and on the relevant forms and reported to the CI and Sponsor. Deviations should also be reported to the EC as part of the EC's continued review of the Trial (e.g. through the ASR, APR, etc.). Protocol deviations which are found to recur frequently will require (immediate) action. Investigator acknowledges that such recurring protocol deviations could potentially be classified as a severe violation.

It is understood that "a serious violation" is likely to affect to a significant degree:

- the safety or physical or mental integrity of the Trial participants; or
- the scientific validity of the Trial

The Investigator is expected to take any immediate action required to protect the safety of any participant included in the Trial, even if this action represents a deviation from the protocol. In such cases, the Sponsor should be notified of this action, and the EC at the Trial site should be informed according to local procedures and regulations.

8.4 Data protection and participant confidentiality

The Trial will be conducted in compliance with the requirements of the EU General Data Protection Regulation 2016/679 (GDPR), the relevant Belgian laws implementing the GDPR including the Belgian Privacy Act of 30 July 2018 on the protection of privacy in relation to the processing of personal data. Any collection, processing and disclosure of personal data, such as participant health and medical information is subject to compliance with the aforementioned personal data protection laws (cfr. Data

Processing Annex (DPA) in Appendix). In case personal data is transferred outside the European Economic Area, safeguards will be taken to ensure that appropriate protection travels with the data in accordance with the GDPR. (https://ec.europa.eu/info/law/law-topic/data-protection/international-dimension-data-protection/rules-international-data-transfers_en#documents)

Any personal data shall be treated as confidential at all times including during collection, handling and use or processing, and the personal data (including in any electronic format) shall be stored securely at all times and with all technical and organizational security measures that would be necessary for compliance with EU and national data protection legislation (whichever is more stringent). The Sponsor shall take appropriate measures to ensure the security of all personal data and guard against unauthorized access thereto or disclosure thereof or loss or destruction while in its custody.

8.5 Insurance

The Participating Site, the Investigator and Sponsor shall have and maintain in full force and effect during the term of this Trial, and for a reasonable period following termination of the Trial, adequate insurance coverage for: (i) medical professional and/or medical malpractice liability, and (ii) general liability.

For Belgian Participating Sites

Art 29 of the Belgian Law relating to experiments on human persons dated May 7th, 2004 applies.
Prior to the start of the Trial, the Sponsor shall enter into an insurance contract in order to adequately cover Trial participants from Belgian sites in accordance with art. 29 of the said law.

For non-Belgian Participating Sites

The Participating Site shall have and maintain in full force and effect during the term of this Trial (and for a reasonable period following termination of the Trial, adequate insurance coverage for other possible damages resulting from the Trial at the Participating Site, as required by local law. Each such insurance coverage shall be in amounts appropriate to the conduct of the services of the Participating Site under this Trial. The Participating Site and Sponsor shall be solely responsible for any deductible or self-insured retention under any such policies.

8.6 Amendments

Unless for urgent reasons as specified in ICH-GCP E6(R2) section 4.5.4, amendments must not be implemented prior to EC and/or CA review and/or approval, as applicable.
In accordance with the Belgian law of May 7th 2004 regarding experiments on humans, the Sponsor may develop a non-substantial amendment at any time during the Trial. If a substantial amendment to the clinical Trial agreement or the documents that supported the original application for the clinical Trial authorisation is needed, the Sponsor must submit a valid substantial amendment to the Competent Authority (CA) for consideration, and to the EC for review and approval. The CA and/or EC will provide a response in accordance with timelines defined by applicable regulations. It is the Sponsor's responsibility to assess whether an amendment is substantial or non-substantial for the purpose of submission to the CA and/or EC.

Amendments to the Trial are regarded as 'substantial' when they are likely to have a significant impact on the safety or physical or mental integrity of the clinical Trial participants, or the scientific value of the Trial.

https://ec.europa.eu/health/sites/health/files/files/eudralex/vol-10/2010_c82_01/2010_c82_01_en.pdf

8.7 Post-Trial activities

Not applicable.

9 Research Registration, Dissemination of Results and Publication Policy

The Declaration of Helsinki (latest version) and European and Belgian regulations require that every research Trial involving human participants be registered in a publicly accessible database before recruitment of the first participant. The CI is responsible for registering the Trial.

In addition, the CI will fulfill their ethical obligation to disseminate and make the research results publicly available. As such, the CI is accountable for the timeliness, completeness, and accuracy of the reports. Researchers, authors, Sponsors, editors, and publishers must adhere to accepted guidelines for ethical reporting. Negative and inconclusive, as well as positive results, must be published or otherwise made publicly available. Sources of funding, institutional affiliations, and conflicts of interest must be declared in the publication.

Publications will be coordinated by the CI. Authorship to publications will be determined following the requirements published by the International Committee of Medical Journal Editors and following the requirements of the respective medical journal.

For multi-centric Trials, it is anticipated that the primary results of the overall Trial shall be published in a multi-center publication.

Participating Sites are not allowed to publish any subset data or results from the Trial before such a multicentre publication.

No information, data, or results of the study may be used for publication without the written approval of the project team. All the members of the project team and all those who contribute in a substantial way to the results of the study and/or the writing of the paper will be the authors of a publication (manuscript, article, abstract, or oral presentation). The first author will be the person who writes the (full journal) paper or who presents the results at a conference (abstract/oral presentation).

Any publication by a Participating Site must be submitted to the Sponsor for review at least thirty (30) calendar days before submission or disclosure. The Sponsor shall have the right to delay the projected publication for a period of up to three (3) months from the date of the first submission to the Sponsor in order to enable the Sponsor to take steps to protect its intellectual property rights and know-how.

10 Intellectual Property

Any know-how, inventions, methods, developments, innovations, discoveries, and therapies, whether patentable or not, arising from the Trial or made in the performance of the Trial protocol ("Inventions") shall vest in the Sponsor. The Participating Site, its employees, and Investigator(s) shall promptly disclose to the Sponsor any such Inventions. Parties have expressly agreed that any Trial data as collected and prepared in the performance of the Trial protocol shall be the sole property of the Sponsor. Publication policy guidelines will be created.

11 Joint Commission International (JCI)

In order to ensure the same quality and safety standards inpatient care for clinical research as commonly applied by the Sponsor in its regular activities, and following JCI standards, the Sponsor shall comply with the following obligations: (a) the Sponsor will use trained and qualified employees or contractors to manage and coordinate the Trial; (b) the Sponsor will ensure that multi-center Trial reporting is reliable and valid, statistically accurate, ethical, and unbiased. (c) the Sponsor will not grant incentives, other than standard compensations and reimbursement of costs, to Trial participants or to participating site's staff that would compromise the integrity of the research; (d) the Sponsor is responsible for monitoring and evaluating the quality, safety, and ethics of the Trial and will respect the participating site's policies and processes when performing such monitoring and evaluation activities; (e) the Sponsor will protect the privacy and confidentiality of the Trial participants following all applicable laws.

12 References

1. Zhou, Yu, Du,*et al.* Clinical course and risk factors for mortality of adult inpatients with COVID-19 in Wuhan, China: a retrospective cohort study. Lancet 2020;395
2. Keyaerts, Vijgen, Maes,*et al.* In vitro inhibition of severe acute respiratory syndrome coronavirus by chloroquine. Biochem Biophys Res Commun 2004;323
3. Zhou, Zhang, Tian,*et al.* Risk factors associated with disease progression in a cohort of patients infected with the 2019 novel coronavirus. Ann Palliat Med 2020
4. Ruan, Yang, Wang,*et al.* Clinical predictors of mortality due to COVID-19 based on an analysis of data of 150 patients from Wuhan, China. Intensive Care Med 2020
5. Lillicrap. Disseminated intravascular coagulation in patients with 2019-nCoV pneumonia. J Thromb Haemost 2020;18
6. Tang, Li, Wang,*et al.* Abnormal coagulation parameters are associated with poor prognosis in patients with novel coronavirus pneumonia. J Thromb Haemost 2020;18
7. Wu, Chen, Cai,*et al.* Risk Factors Associated With Acute Respiratory Distress Syndrome and Death in Patients With Coronavirus Disease 2019 Pneumonia in Wuhan, China. JAMA Intern Med 2020
8. Mehta, McAuley, Brown,*et al.* COVID-19: consider cytokine storm syndromes and immunosuppression. Lancet 2020;395
9. Ji, Zhao, Matalon,*et al.* Elevated Plasmin(ogen) as a Common Risk Factor for COVID-19 Susceptibility. Physiol Rev 2020;100
10. Tang, Bai, Chen,*et al.* Anticoagulant treatment is associated with decreased mortality in severe coronavirus disease 2019 patients with coagulopathy. J Thromb Haemost 2020
11. Thachil. The versatile heparin in COVID-19. J Thromb Haemost 2020
12. Vaninov. In the eye of the COVID-19 cytokine storm. Nat Rev Immunol 2020
13. Huang, Wang, Li,*et al.* Clinical features of patients infected with 2019 novel coronavirus in Wuhan, China. Lancet 2020;395
14. Hoffmann, Kleine-Weber, Schroeder,*et al.* SARS-CoV-2 Cell Entry Depends on ACE2 and TMPRSS2 and Is Blocked by a Clinically Proven Protease Inhibitor. Cell 2020
15. Vaduganathan, Vardeny, Michel,*et al.* Renin-Angiotensin-Aldosterone System Inhibitors in Patients with Covid-19. N Engl J Med 2020
16. Brisse, Wouters, Andrei,*et al.* How Viruses Contribute to the Pathogenesis of Hemophagocytic Lymphohistiocytosis. Front Immunol 2017;8
17. Brisse, Wouters, Matthys. Hemophagocytic lymphohistiocytosis (HLH): A heterogeneous spectrum of cytokine-driven immune disorders. Cytokine Growth Factor Rev 2015;26
18. H, I, H,*et al.* Chemistry and Biochemistry of Proteinase Inhibitors From Mammalian Tissues. Annals of the New York Academy of Sciences 1968;146
19. E, I, H,*et al.* Physiologic, Pharmacologic, and Clinicalspects of Proteinase Inhibitors. Annals of the New York Academy of Sciences 1968;146
20. H, G. Biochemistry and Applications of Aprotinin, the Kallikrein Inhibitor From Bovine Organs. Arzneimittel-Forschung 1983;33
21. Fritz, Wunderer. Biochemistry and Applications of Aprotinin, the Kallikrein Inhibitor from Bovine Organs. Arzneimittelforsch 1983;33-1
22. Fritz, Trautschold, Haendle,*et al.* Chemistry and Biochemistry of Proteinase Inhibitors from Mammalian Tissues. Ann Ny Acad Sci 1968;146
23. R, R. Aprotinin. A Review of Its Pharmacology and Therapeutic Efficacy in Reducing Blood Loss Associated With Cardiac Surgery. Drugs 1995;49
24. KS. Aprotinin Dosing: How Much Is Enough? The journal of extra-corporeal technology 2004;36
25. T, Y, S,*et al.* Treatment of Acute Pancreatitis With Protease Inhibitors Administered Through Intravenous Infusion: An Updated Systematic Review and Meta-Analysis. BMC gastroenterology 2014;14
26. D, BP, KM,*et al.* Effect of Aprotinin on Need for Blood Transfusion After Repeat Open-Heart Surgery. Lancet (London, England) 1987;2
27. W, NJ, BP,*et al.* Effects of Aprotinin on Hemostatic Mechanisms During Cardiopulmonary Bypass. The Annals of thoracic surgery 1987;44
28. C, M, W. Feasibility Study of Very High Aprotinin Dosage in Polytrauma Patients. Progress in clinical and biological research 1987;236A
29. JH, JM, M. Pharmacokinetics of Aprotinin in Preoperative Cardiac Surgical Patients. Anesthesiology 1994;80
30. H, M, O,*et al.* Aprotinin Concentrations Effective for the Inhibition of Tissue Kallikrein and Plasma Kallikrein in Vitro and in Vivo. Advances in experimental medicine and biology 1989;247B

31. Llitjos, Leclerc, Chochois,*et al.* High incidence of venous thromboembolic events in anticoagulated severe COVID-19 patients. J Thromb Haemost 2020
32. Eloseily, Weiser, Crayne,*et al.* Benefit of Anakinra in Treating Pediatric Secondary Hemophagocytic Lymphohistiocytosis. Arthritis Rheumatol 2020;72
33. Aytac, Batu, Unal,*et al.* Macrophage activation syndrome in children with systemic juvenile idiopathic arthritis and systemic lupus erythematosus. Rheumatol Int 2016;36
34. Ravelli, Grom, Behrens,*et al.* Macrophage activation syndrome as part of systemic juvenile idiopathic arthritis: diagnosis, genetics, pathophysiology and treatment. Genes Immun 2012;13
35. Chen, Wu, Guo,*et al.* Clinical and immunologic features in severe and moderate Coronavirus Disease 2019. J Clin Invest 2020
36. Galea, Ogungbenro, Hulme,*et al.* Intravenous anakinra can achieve experimentally effective concentrations in the central nervous system within a therapeutic time window: results of a dose-ranging study. J Cerebr Blood F Met 2011;31
37. Opal, Fisher, Dhainaut,*et al.* Confirmatory interleukin-1 receptor antagonist trial in severe sepsis: a phase III, randomized, double-blind, placebo-controlled, multicenter trial. The Interleukin-1 Receptor Antagonist Sepsis Investigator Group. Crit Care Med 1997;25
38. Fisher, Slotman, Opal,*et al.* Initial evaluation of human recombinant interleukin-1 receptor antagonist in the treatment of sepsis syndrome: a randomized, open-label, placebo-controlled multicenter trial. Crit Care Med 1994;22
39. Granowitz, Porat, Mier,*et al.* Pharmacokinetics, safety and immunomodulatory effects of human recombinant interleukin-1 receptor antagonist in healthy humans. Cytokine 1992;4

APPENDICES

13 Appendix 1: Data Processing Annex (DPA)

Definitions:

- "Protocol" means the document entitled "A randomized, open-label, adaptive, proof-of-concept clinical trial of modulation of the thromboinflammatory response in patients with COVID-19" containing the details of the academic Trial as developed by the Sponsor and approved by the relevant Ethics Committee.
- "Sponsor" means University Hospitals Leuven (UZ Leuven).
- Participating site acts as a data processor as defined under article 4, 8) of the Regulation (EU) 2016/679 ("Data Processor") for the Sponsor who acts as a data controller as defined under article 4, 7) of the Regulation (EU) 2016/679 ("Data Controller").
- "Applicable Law" means any applicable data protection or privacy laws, including:
  a) the Regulation (EU) 2016/679 also referred to as the General Data Protection Regulation ("GDPR");
  b) other applicable laws that are similar or equivalent to or that are intended to or implement the laws that are identified in (a) of this definition;
- "Personal Data" means any information relating to an identified or identifiable natural person ("Data Participant"), including without limitation pseudonymized information, as defined in Applicable Law and described in the Protocol.

Rights and obligations:

1. The Data Processor is instructed to process the Personal Data for the term of the Trial and only to provide the data processing tasks set out in the Protocol. The Data Processor may not process or use Personal Data for any purpose other than a Data Participant's medical records, or other than provided in the instructions of the Trial protocol, including concerning transfers of personal data to a third country or an international organization unless the Data Processor is required to do so according to Union or Member State law.
2. Data Processor shall at all times maintain a record of processing of Personal Data Applicable Law and if the Data Processor considers an instruction from the Data Controller to be in violation of the Applicable Law, the Data Processor shall promptly inform the Data Controller in writing about this.
3. The Data Processor must ensure that persons authorized to process the Personal Data have committed themselves to confidentiality or are under an appropriate statutory obligation of confidentiality.
4. The Data Processor shall implement appropriate technical and organizational measures to prevent that the Personal Data processed is:
   (i) accidentally or unlawfully destroyed, lost or altered,
   (ii) disclosed or made available without authorization, or
   (iii) otherwise processed in violation of Applicable Law.
5. The appropriate technical and organizational security measures must be determined with due regard for:
   (i) the current state of the art,
   (ii) the cost of their implementation, and

(iii) the nature, scope, context and purposes of processing as well as the risk of varying likelihood and severity for the rights and freedoms of natural persons.

6. Taking into account the nature of the processing, the Data Processor shall assist the Data Controller, by means of appropriate technical and organizational measures, insofar as this is possible, in fulfilling its obligation to respond to requests from Data Participants pursuant to laws and regulations in the area of privacy and data protection (such as, the right of access, the right to rectification, the right to erasure, the right to restrict the processing, the right to data portability and the right to object)

7. The Data Processor shall upon request provide the Data Controller with sufficient information to enable the Data Controller to ensure that the Data Processor's obligations under this DPA are complied with, including ensuring that the appropriate technical and organizational security measures have been implemented.

8. The Data Controller is entitled to appoint at its own cost an independent expert, reasonably acceptable to the Data Processor, who shall have access to the Data Processor's data processing facilities and receive the necessary information for the sole purpose of auditing whether the Data Processor has implemented and maintained said technical and organizational security measures. The expert shall upon the Data Processor's request sign a non-disclosure agreement provided by the Data Processor, and treat all information obtained or received from the Data Processor confidentially, and may only pass on, after conferral with the Data Processor, the findings as described under 10) (ii) below to the Data Controller.

9. The Data Processor must give authorities who by Union or Member State law have a right to enter the Data Controller's or the Data Controller's processors' facilities, or representatives of the authorities, access to the Data Processor's physical facilities against proper proof of identity and mandate, during normal business hours and upon reasonable prior written notice.

10. The Data Processor must without undue delay in writing notify the Data Controller about:

    (i) any request for disclosure of Personal Data processed under the Protocol by authorities, unless expressly prohibited under Union or Member State law,

    (ii) any finding of (a) breach of security that results in accidental or unlawful destruction, loss, alteration, unauthorized disclosure of, or access to, Personal Data transmitted, stored or otherwise processed by the Data Processor under the Protocol, or (b) other failure to comply with the Data Processor's obligations, or

    (iii) any request for access to the Personal Data (with the exception of medical records for which the Data Processor is considered data controller) received directly from the Data Participants or from third parties.

11. Such a notification from the Data Processor to the Data Controller with regard to a breach of security as meant in 10) (ii)(a) above will contain at least the following information:

    (i) the nature of the Personal Data breach, stating the categories and (by approximation) the number of Data Participants concerned, and stating the categories and (by approximation) the number of the personal data registers affected (datasets);

    (ii) the likely consequences of the Personal Data breach;

    (iii) a proposal for measures to be taken to address the Personal Data breach, including (where appropriate) measures to mitigate any possible adverse effects of such breach.

12. The Data Processor shall document (and shall keep such documentation available for the Data Controller) any Personal Data breaches, including the facts related to the Personal Data breach, its effects and the corrective measures taken. After consulting with the Data Controller, the Data Processor shall take any measures needed to limit the (possible) adverse effects of Personal Data breaches (unless such consultation cannot be awaited due to the nature of the Personal Data breach).

13. The Data Processor must promptly and reasonably assist the Data Controller (with the handling of (a) responses to any breach of security as described in 10) (ii) above and (b) any requests from

Data Participants under Chapter III of the GDPR, including requests for access, rectification, blocking or deletion. The Data Processor must also reasonably assist the Data Controller by implementing appropriate technical and organizational measures for the fulfilment of the Data Controller's obligation to respond to such requests.

14. The Data Processor must reasonably assist the Data Controller with meeting the other obligations that may be incumbent on the Data Controller according to Union or Member State law where the assistance of the Data Processor is implied, and where the assistance of the Data Processor is necessary for the Data Controller to comply with its obligations. This includes, but is not limited to, at the request to provide the Data Controller with all necessary information about an incident under 10) (ii), and all necessary information for an impact assessment in accordance with Article 35 and Article 36 of the GDPR.

Subprocessor:

15. The Data Processor may only engage a subprocessor, with prior specific or general written consent from the Data Controller. The Data Processor undertakes to inform the Data Controller of any intended changes concerning the addition or replacement of a subprocessor by providing a reasonable prior written notice to the Data Controller. The Data Controller may reasonably and in a duly substantiated manner object to the use of a subprocessor. The Data Processor must inform the Data Controller in writing of the discontinued use of a subprocessor.
16. Prior to the engagement of a subprocessor, the Data Processor shall conclude a written agreement with the subprocessor, in which at least the same data protection obligations as set out in this DPA shall be imposed on the subprocessor, including obligations to implement appropriate technical and organizational measures and to ensure that the transfer of Personal Data is done in such a manner that the processing will meet the requirements of the Applicable Law.
17. The Data Controller has the right to receive a copy of the relevant provisions of Data Processor's agreement with the subprocessor related to data protection obligations. The Data Processor shall remain fully liable to the Data Controller for the performance of the subprocessor obligations under this DPA. The fact that the Data Controller has given consent to the Data Processor's use of a subprocessor is without prejudice for the Data Processor's duty to comply with this DPA.
